# Regulating Adsorption Behaviors in Crystalline Porous Materials by External Electric Fields

**DOI:** 10.1002/smsc.202400391

**Published:** 2025-01-03

**Authors:** Yang Yang, Tianyi Zhang, Tianqi Wang, Teng Zhou, Youssef Belmabkhout, Qinfen Gu, Jin Shang

**Affiliations:** ^1^ School of Energy and Environment City University of Hong Kong Tat Chee Avenue Kowloon Hong Kong 999077 China; ^2^ Sustainable Energy and Environment Thrust The Hong Kong University of Science and Technology (Guangzhou) Nansha Guangzhou 511400 China; ^3^ Applied Chemistry and Engineering Research Centre of Excellence (ACER CoE) Mohammed VI Polytechnic University (UM6P) Lot 660 – Hay Moulay Rachid Ben Guerir 43150 Morocco; ^4^ Australian Synchrotron ANSTO 800 Blackburn Road Clayton VIC 3168 Australia

**Keywords:** adsorptions, crystalline porous materials, electric fields

## Abstract

The regulation of adsorption behaviors in crystalline porous materials (CPMs) using external electric fields (E‐fields) is an emerging field. CPMs are highly valued for their large surface area, well‐ordered pore structures, and chemical versatility, making them ideal for applications in adsorption/separation, catalysis, and biomedicine. In this review, the mechanisms underlying the E‐field‐induced structural and functional modifications in CPMs, such as phase transitions, framework distortions, and alterations in pore accessibility and selectivity, are delved into. Through a comprehensive analysis, the adsorption behaviors influenced by E‐field regulation are classified into three main categories: selective molecular adsorption, selective ion adsorption, and the diffusion/transportation of molecules and ions. Furthermore, in this review, the current landscape of research in this area, highlighting the challenges and future directions for developing E‐field‐regulated adsorbents is critically assessed. In this work, it is aimed to summarize recent advances and identify gaps in the understanding of E‐field effects on CPMs, providing a foundation for the rational development of next‐generation materials with E‐field modulated functionalities.

## Graphical abstract

Applying external electric field can regulate the adsorption behaviors of crystalline porous materials via inducing their structural and functional modifications, opening the avenue for many promising applications.

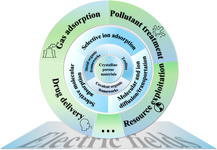

## 1 Introduction

Crystalline porous materials (CPMs), such as zeolites,^[^ ^1^ ^]^ metal‐organic frameworks (MOFs),^[^ ^2^ ^]^ and covalent organic frameworks (COFs),^[^ ^3^ ^]^ are a class of versatile materials with regularly arranged pore structures. These materials exhibit remarkable physical and chemical properties, such as large surface areas, well‐ordered micro/mesoporous crystalline structures, selective adsorption capabilities, and tunable catalytic activities. Their superiorities of readily adjustable porous structures and functionalities have driven intensive research into their design and synthesis for various applications, including molecular/ion adsorption and separation,^[^ ^4^, ^5^, ^6^ ^]^ catalysis,^[^ ^7^ ^]^ and biomedical uses.^[^ ^8^ ^]^ The molecular/ion adsorption behavior plays a central role in some of these applications and thus has attracted considerable research interest over the past decades.

Tuning CPMs from structural or chemical aspects, such as cation doping,^[^ ^9^, ^10^ ^]^ ligand exchange or functionalization,^[^ ^11^, ^12^, ^13^ ^]^ surface modification,^[^ ^14^, ^15^ ^]^ and framework interpenetration,^[^ ^16^, ^17^ ^]^ have been developed to enhance their performance. Moreover, external stimuli, such as guest accommodation,^[^ ^18^, ^19^, ^20^, ^21^, ^22^ ^]^ thermal stimulation,^[^ ^23^, ^24^, ^25^, ^26^ ^]^ light,^[^ ^27^, ^28^, ^29^ ^]^ pressure,^[^ ^30^, ^31^ ^]^ electric fields (E‐field),^[^ ^32^, ^33^, ^34^ ^]^ and magnetic fields,^[^ ^35^, ^36^ ^]^ have been intensively used to regulate the structure and properties of CPMs, thereby tuning the adsorption characteristics of these materials. Among them, E‐field shows great promise due to their ease of application, low energy consumption, rapid response, and precise control.^[^ ^37^, ^38^, ^39^, ^40^, ^41^, ^42^, ^43^, ^44^, ^45^, ^46^, ^47^, ^48^ ^]^ In particular, MOFs and zeolites have been intensively studied for their response to E‐fields, demonstrating potential in electrical conductivity regulation, structural tuning, dielectric property adjustments, and enhanced selective adsorption, separation, diffusion, transport, and catalytic performance.^[^ ^33^, ^37^, ^49^, ^50^, ^51^, ^52^, ^53^, ^54^, ^55^, ^56^, ^57^, ^58^, ^59^, ^60^, ^61^, ^62^ ^]^ When subjected to an external E‐field, CPMs could undergo phase transitions, volume changes, framework deformations, local rotations of dipolar groups, or movements of gate‐keeping ions, thus altering pore accessibility and adsorption affinity.^[^ ^37^, ^38^, ^39^, ^40^, ^41^, ^42^, ^43^, ^44^, ^45^, ^46^, ^47^, ^48^ ^]^ Understanding how external E‐fields can modulate CPMs to regulate their physicochemical properties and the consequent adsorption behaviors is crucial for making use of this stimulus for achieving desirable adsorption performance.

While the focus of our discussion has been on the well‐ordered structures of CPMs, it is also worthwhile to briefly consider the realm of amorphous porous materials. These materials, with their unique characteristics, provide an interesting contrast. They exhibit irregular pore sizes and shapes, resulting in structural heterogeneity that leads to localized E‐field‐induced effects. Theoretically, the response of these materials to an E‐field is more uneven and unpredictable compared to CPMs. Unlike hydrogels or activated carbon, which have been extensively studied for their E‐field‐induced adsorption properties in medical and environmental applications due to their excellent electrical conductivity,^[^ ^63^, ^64^, ^65^ ^]^ the application of E‐fields to CPMs has only recently gained significant interest as a stimulus. However, further research is required to fully understand the behavior of CPMs under E‐field, particularly in comparison to amorphous porous materials. Despite an increase in academic research encompassing both theoretical studies, such as molecular dynamics (MD) simulations, and experimental investigations, there remains a gap in the systematic understanding of how E‐fields influence the adsorption behavior of CPMs. A comprehensive literature review is essential to integrate key research insights and identify major trends in this field.

**Figure 1:**
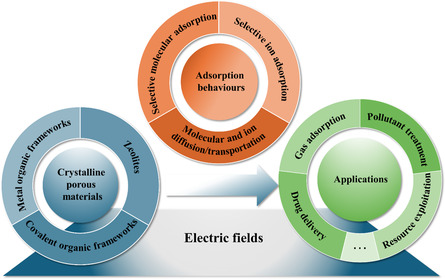
Schematic illustration of E‐field‐regulated CPMs and their adsorption behaviors and applications.

The objective of the review is to summarize recent advances in the use of external E‐fields to regulate the behavior of CPMs. We started by discussing the E‐field‐induced changes in CPMs, focusing on phase transition, breathing behavior, electric properties, and charge transfer, which serve as the foundation for understanding subsequent applications of E‐field regulation. We then evaluated the effect of external E‐fields on the applications of CPMs. When exposed to an E‐field, CPMs can be used in conjunction with membranes or other materials, allowing for the adsorption, separation, and storage/release of molecules through the regulation of E‐fields **(** **Figure** **1**). In solution, CPMs can adsorb and separate ions under the influence of an E‐field. Additionally, under an E‐field, the restrictive pores of CPMs can serve as transport channels for certain ions and molecules. We classified the effects of external E‐field on the adsorption applications of CPMs into three main categories: 1) selective molecular adsorption, 2) selective ion adsorption, and 3) molecular and ion diffusion/transportation. Finally, we assessed the current state of research and explored the challenges and future directions in developing materials using E‐field regulations. Notably, while other external stimuli have been well studied and summarized in the literature, there is a significant lack of systematic analysis in the literature on the regulation of adsorption processes in CPMs using E‐field. This review aims to address this gap and serves as a vital theoretical framework for the rational development of CPMs with tunable performance under external E‐fields.

## 2 E‐Field‐Induced Changes in CPMs

The properties of CPMs are closely related to chemical compositions and chemical bonding of the porous structure. These materials can undergo structural transformations accompanied by alterations in electrical properties when subjected to external stimuli, leading to significant changes in their properties and performance. Such transformations include phenomena like pore breathing and reversible phase transitions, which are particularly notable in flexible frameworks such as MOFs and COFs, distinguished from rigid frameworks. Additionally, even zeolites, typically regarded as rigid, can demonstrate electro‐responsive behaviors under certain conditions. The well‐defined structures, exceptional tunability, high surface areas, and broad application potential of these materials make them ideal candidates for exploring electro‐responsive behavior as a handle to manipulate material performance. Here, we provide a detailed overview of the E‐field‐induced changes in three types of CPMs (i.e., zeolite, MOFs, and COFs), and how these changes can influence their adsorption behavior when an E‐field is applied.

### 2.1 Zeolites

Zeolites are microporous crystalline aluminosilicates, built from linked SiO_4_^−^ and AlO_4_^−^ tetrahedra, forming 3D framework structures.^[^ ^1^ ^]^ Their unique microporous structures and high specific surface areas enable a strong adsorption capability toward various small molecules and ions. A unique characteristic of zeolites is the presence of exchangeable, mobile cations (e.g., Na^+^, K^+^, Ca^2+^, and Mg^2+^) located on the internal surface of the nanopores. These cations compensate for the charge deficit resulting from the substitution of Al to Si in the SiO_2_ hosting framework.^[^ ^1^ ^]^ Consequently, the conductivity of zeolites is predominantly associated with ionic conductivity. In addition, the surface of zeolites is usually negatively charged and can trap cations effectively, enabling ion‐exchange processes. An applied external E‐field can affect zeolite structure thorough framework expansion and ions relocation.^[^ ^37^ ^]^ This E‐field can also alter charge‐transport mechanisms in zeolite based on the ionic conductivity characteristics.^[^ ^38^, ^39^, ^66^ ^]^

**Figure 2:**
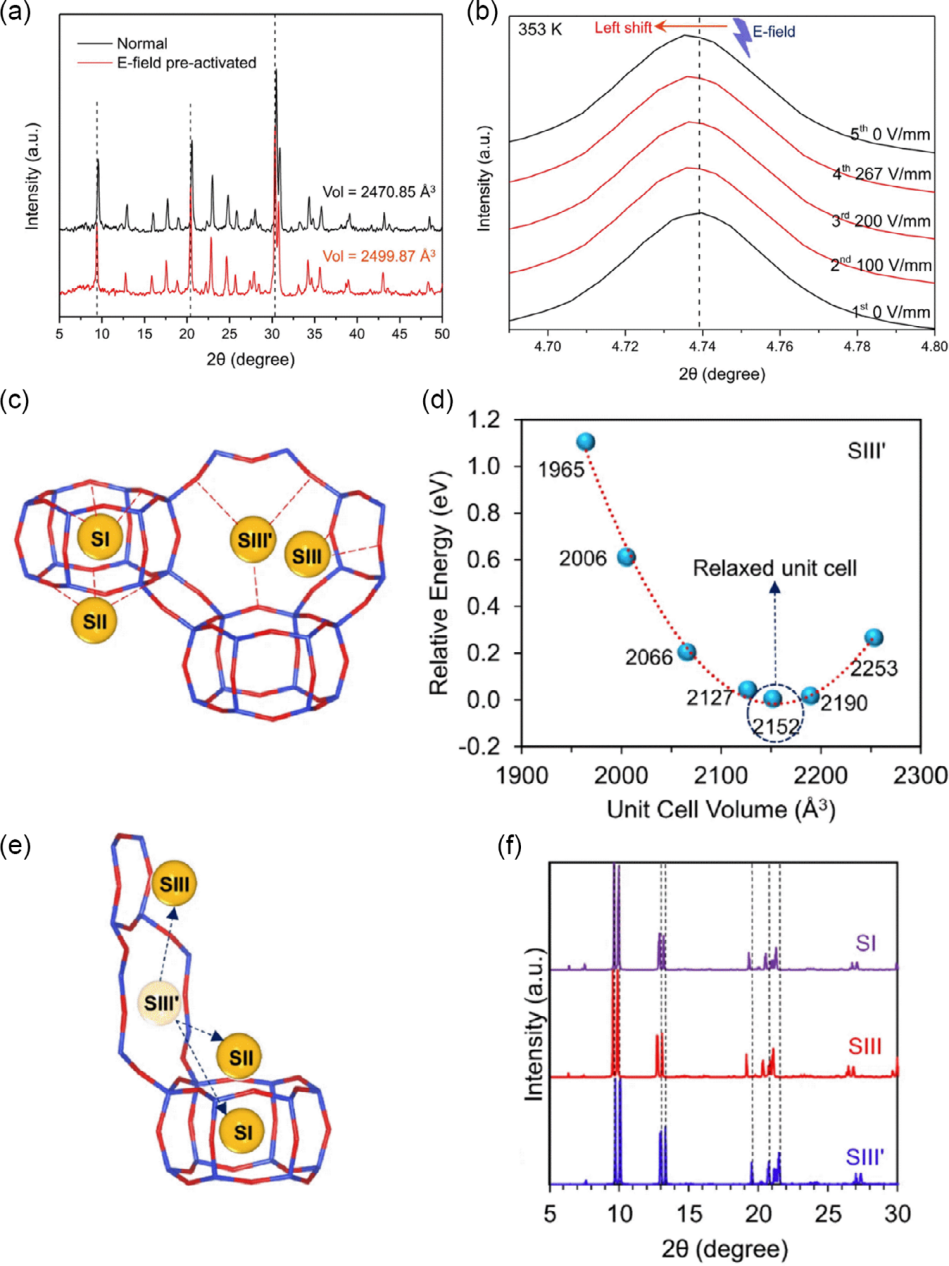
a) PXRD patterns of r2KCHA before and after the E‐field pre‐activation. b) PXRD patterns of r2KCHA with in situ E‐field application at 353 K. c) The schematic illustration of chabazite with four cation sites. Yellow: potassium, blue: silicon or aluminum, and red: oxygen. d) Changes of the relative energy over unit cell volume with all K^+^ located at SIII′. e) The pathways for the K^+^ relocating from SIII′ to other cation sites. f) The simulated PXRD patterns of r2KCHA unit cell with eight K^+^ ions located at SIII′ site and one K^+^ ion located at SI, SIII and SIII′ site, respectively. Reproduced with permission.^[^ ^37^ ^]^ Copyright 2023, Springer Nature.

E‐field pre‐activation of zeolites could induce migration of extra‐framework cations and framework expansion. Li and coworkers reported a study on zeolite pre‐activation by external E‐field, revealing an E‐field‐induced structural transition.^[^ ^37^ ^]^ It has been well acknowledged that zeolites frameworks lack moveable linkages or functional groups, leading to limited relaxation under pressure‐induced hydration or varying temperature. Considerable changes in the unit cell parameters are always accompanied by cation relocation.^[^ ^67^ ^]^ The powder X‐Ray diffraction (PXRD) patterns of E‐field pre‐activated chabazite (r2KCHA) indicated a framework expansion (**Figure** **2**a). Synchrotron PXRD analysis further suggested a sustainable effect induced by E‐field, as shown in Figure 2b. The 3D structure of chabazite constitutes a large supercavity accessed by six eight‐membered rings (8MR), with four general cation positions (Figure 2c). Density‐functional theory (DFT) calculations showed that when all cations are located at SIII' site, the relative energy reaches the minimum, and the unit cell volume of r2KCHA is at its smallest (2152 Å^3^), as shown in Figure 2d. The unit cell expands if K^+^ relocates away from the original SIII′ site. Additionally, the increase of BET surface area after the E‐field pre‐activation further verifies the cation relocation in r2KCHA. This study represents the combined experimental and computational investigation of E‐field‐induced structural transformation of zeolite, showing implications for understanding the molecular sieving capability and adsorption properties of zeolite.

The charge‐transport properties of zeolites are significantly influenced by external E‐field. Salamov research group has conducted a series studies on the electronic and ionic‐transport mechanisms of nanoporous clinoptilolite by electrical characterizations.^[^ ^38^, ^39^, ^66^ ^]^ The applied E‐field influenced the ion migration, facilitated by lattice defects (vacancies and interstitials) in the structure, thereby allowing for determining the characteristics of the discharge plasma and charge‐transport mechanisms. Applying an E‐field of 2 kV cm^−1^ strength for 240 min resulted in a sharp enhancement in transport property.^[^ ^66^ ^]^ It was inferred that E‐field produced a polarization within zeolite and thus promoted ion movement. When a relative high voltage of 60‐200 kV cm^−1^ was applied, ionization occurred as the E‐field reached the breakdown voltage.^[^ ^38^ ^]^ At this stage, electronic conduction contributed to the overall conduction process within the zeolite. Moreover, it was discovered that zeolite modified by silver ion exchange could effectively reduce the breakdown voltage, enabling charge transport at lower voltages.^[^ ^39^ ^]^ Zeolites with high conductivity have been shown to facilitate transport of high‐valence cations more readily. This contributes to a fundamental understanding of ion‐transport/conduction properties in zeolite under E‐field. In addition, cations within the nanopores can interact strongly with water, thereby influencing water adsorption under an E‐field. This phenomenon is significant for studies on ion exchange^[^ ^68^ ^]^ and ion separations by zeolite membranes through electrosorption.^[^ ^69^, ^70^, ^71^ ^]^

The external E‐field can modify the electronic properties of semiconductive zeolites, including the Fermi surface and bandgap, which has implications for their catalytic applications.^[^ ^72^, ^73^ ^]^ For instance, in the case of hydrogen form Zeolite Socony Mobil‐5 (HZSM‐5) used for catalytic olefin production, it was observed that the zeolites remained stable after the reaction when subjected to an external direct‐current E‐field. When an E‐field is applied to the catalyst surface during the reaction, the surface charge increases, causing curvature in the energy band. This leads to an increase in the Fermi level, resulting in a higher yield of olefins. Increasing the input electrical current elevates the number of electrons in the reaction media and thus promotes the reaction. Moreover, the high energy of accelerating electrons in the E‐field can break existing chemical bonds and form new bonds, thereby increasing the number of active sites in zeolites. The bandgap of HZSM‐5 zeolites was found to enhance the surface adsorption of electrophilic oxygen species. Moreover, the E‐field can enhance the catalytic activity of HZSM‐5 by facilitating the adsorption of reactants on the catalyst. Furthermore, the selectivity of zeolite can be influenced by synergetic effect between the electrostatic field within nanopores and the external E‐field.^[^ ^73^ ^]^

### 2.2 MOFs

MOFs have garnered significant interest over the past two decades as a class of crystalline hybrid porous materials. MOFs are constructed from metal ions or metal cluster nodes and multitopic organic linkers with precise spatial control. One key feature of MOFs is their flexibility, which sets them apart from traditional porous materials, such as zeolites. This flexibility allows their deformable periodic frameworks to respond more readily and remarkably to external E‐field. E‐field stimulation often has a significant impact on the properties of MOFs. Therefore, a fundamental understanding of structural evolution and property variation processes regulated by external E‐field is critical for developing applications of MOFs under E‐field.

Upon the application of an E‐field, the linker groups of specific MOFs can experience a rotation process. Winston et al. studied on the E‐field response of MOFs focusing on the rotation of dipolar linker groups.^[^ ^42^ ^]^ Specifically, the polar bromo‐*p*‐phenylene linker in the as‐prepared polar, isoreticular MOFs (IRMOF‐2) experienced a significant angle vibration under an oscillating E‐field, a phenomenon not observed in the nonpolar counterpart, IRMOF‐1. This difference in response to E‐field indicates that modifying the polar diacid linker structure enables lower rotational barrier potential and detectable dipole–dipole interactions in the molecular rotor systems.^[^ ^42^ ^]^ Typically, some of organic linker molecules would be induced and form dipoles within MOF structures and align directionally when an external E‐field is applied, generating an opposite internal E‐field.^[^ ^48^ ^]^ The MD simulations showed that adding rotatable dipolar linkers led to an increased degree of electric response in MOFs.^[^ ^47^ ^]^ A higher degree of dipole moment polarization can reduce the E‐field strength required to trigger responsive behavior of 1D‐type channel Materials of Institute Lavoisier (MIL)‐53(Cr). Likewise, halogenating the rotatable organic linkers of IRMOF‐1 and IRMOF‐7 can create permanent dipole moments, making them responsive to E‐field polarization.^[^ ^74^ ^]^ More importantly, E‐field strength required to control the rotation of linkers can be significantly reduced by altering the size of the ligand, as well as the type, number, and substitution positions of the halogen.

**Figure 3:**
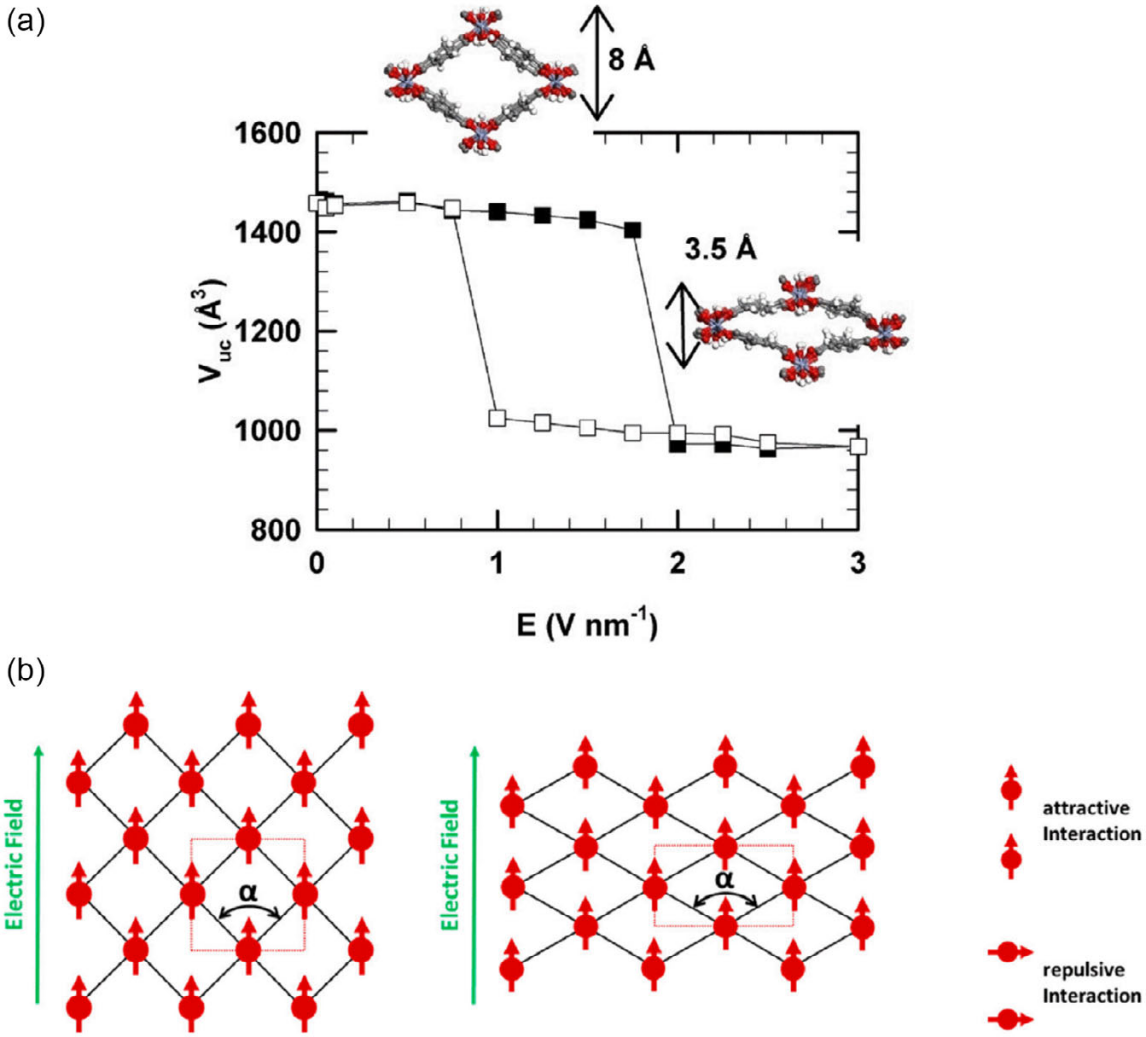
a) For a 2D lattice of induced (aligned) dipoles, the dipole−dipole interaction. Reproduced with permission.^[^ ^57^ ^]^ Copyright 2017, American Chemical Society. b) Unit cell volume of the empty MIL‐53(Cr) as a function of the external electrical field (E). Reproduced with permission.^[^ ^48^ ^]^ Copyright 2017, American Chemical Society.

It has been found that the breathing behavior of MIL‐53 can be triggered by E‐field‐induced dipole–dipole interaction in addition to guest adsorption and thermal stimulus. Ghoufi et al. reported that the MIL‐53(Cr) experienced an electrically induced breathing from the initial large pore (LP) form to the contracted pore form under a 1.75 V nm^−1^ E‐field, causing a unit cell volume change of 35% and a large reduction of the aperture from 12.8 to 7.9 Å, as illustrated in **Figure** **3**a.^[^ ^57^ ^]^ This LP−narrow pore (NP) structural transition is fully reversible with a hysteresis of 1.00 V nm^−1^. It should be noted that the electrical field used in the MD simulations is relatively large with respect to the conventional field strength, which is far above the breakdown voltage of air (≈3 × 10^−3^ V nm^−1^). This is the first observation of an E‐field‐induced breathing effect, but the physical mechanism leading to changes in pore remains unclear. In view of this problem, Schmid proposed that the mutual dipole–dipole interaction is the driving force for breathing behavior, while E‐field is just a necessary trigger based on a theoretical study.^[^ ^48^ ^]^ The increased short‐range attractive interactions and decreased repulsive interactions caused structural deformation into a stabilized rhombic shape, as presented in Figure 3b. Building on this concept, a mathematical illustration of the MOFs’ structural transition under an E‐field was developed using a simplified 2D lattice model,^[^ ^75^ ^]^ demonstrating the polarization density as a function of the applied E‐field. The results are qualitatively consistent with the MD simulations in empty MIL‐53(Cr) despite simplifications. This model can provide the basis for predicting E‐field‐induced structural transitions in MOFs and other materials. Nevertheless, future experiments are necessary to determine the appropriate strength of the E‐field required to induce such a structural transformation.

**Figure 4:**
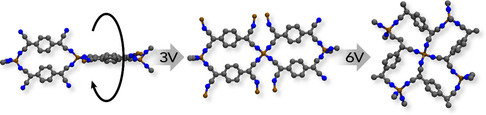
Electric switching of the MOF Cu(TCNQ) from a high‐resistance state to a conducting state by an electric potential. Reproduced with permission.^[^ ^32^ ^]^ Copyright 2014, Springer Nature.

**Figure 5:**
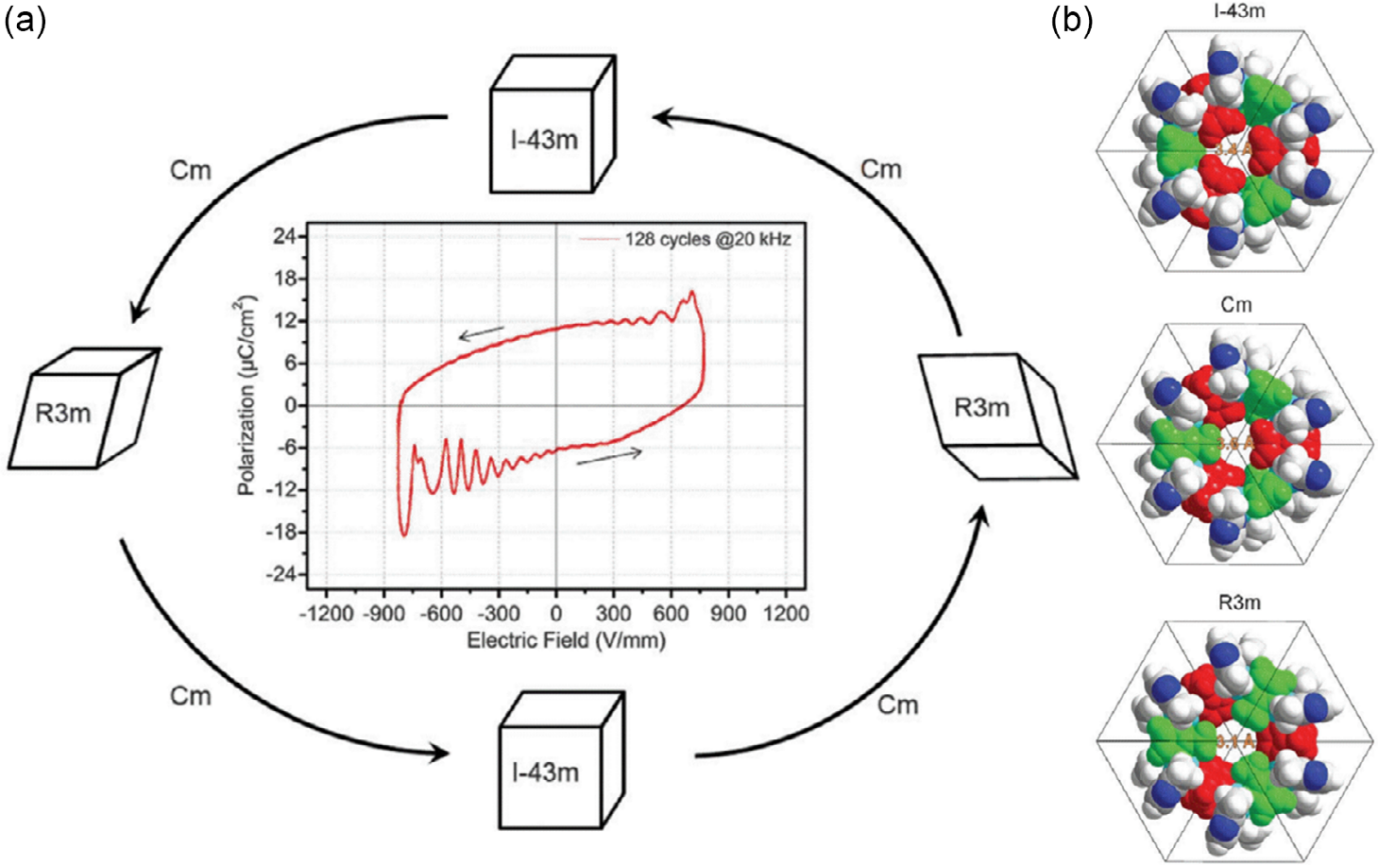
a) Scheme of the structural changes of ZIF‐8 under E‐field. b) A representation of the primitive cells of ZIF‐8 gate as a space‐filling model of different simulated polymorphs. Reproduced with permission. Reproduced with permission.^[^ ^33^ ^]^ Copyright 2017, The American Association for the Advancement of Science.

Another significant response of MOFs to external E‐fields is structural deformation due to phase transitions, driven by the rotation of MOF linkers. As shown in **Figure** **4**, an electrically switchable MOF composed of Cu(TCNQ) (TCNQ: 7,7,8,8‐tetracyanoquinodimethane) transformed from a highly resistive phase to a conductive phase, accompanied by a structural transition. The switching process is fully reversible, with the material reverting to its original insulator phase upon the removal of the applied potential.^[^ ^32^ ^]^ Polycrystalline zeolitic imidazolate framework (ZIF)‐8 also experiences phase transitions when exposed to an external E‐field, thus accurately tuning the flexibility of framework.^[^ ^33^ ^]^ ZIF‐8 underwent a reversible crystal structure transformation from cubic (I$\bar{4}$3m) symmetry to monoclinic (Cm) and triclinic (R3m) polymorphs at higher field (**Figure** **5**). The limiting pore diameter of the Cm phase (the most dominant polarizable phase) is 3.6 Å, slightly larger than that of the pristine cubic ZIF‐8 (3.4 Å). The ab initio DFT calculations also proves that a higher lattice stiffness arises, and the framework fibrillation is reduced by inhibiting linker rotation upon E‐field polarization. Subsequently, it was reported that a ZIF‐8 membrane dominated with stiffened Cm phase can be synthesized directly in an E‐field.^[^ ^54^ ^]^ The space group of the ZIF‐8 lattice changed from I$\bar{4}$3m to Cm during the synthesis process. The ZIF‐8 powder synthesized under E‐field showed a similar transformation.^[^ ^58^ ^]^

**Figure 6:**
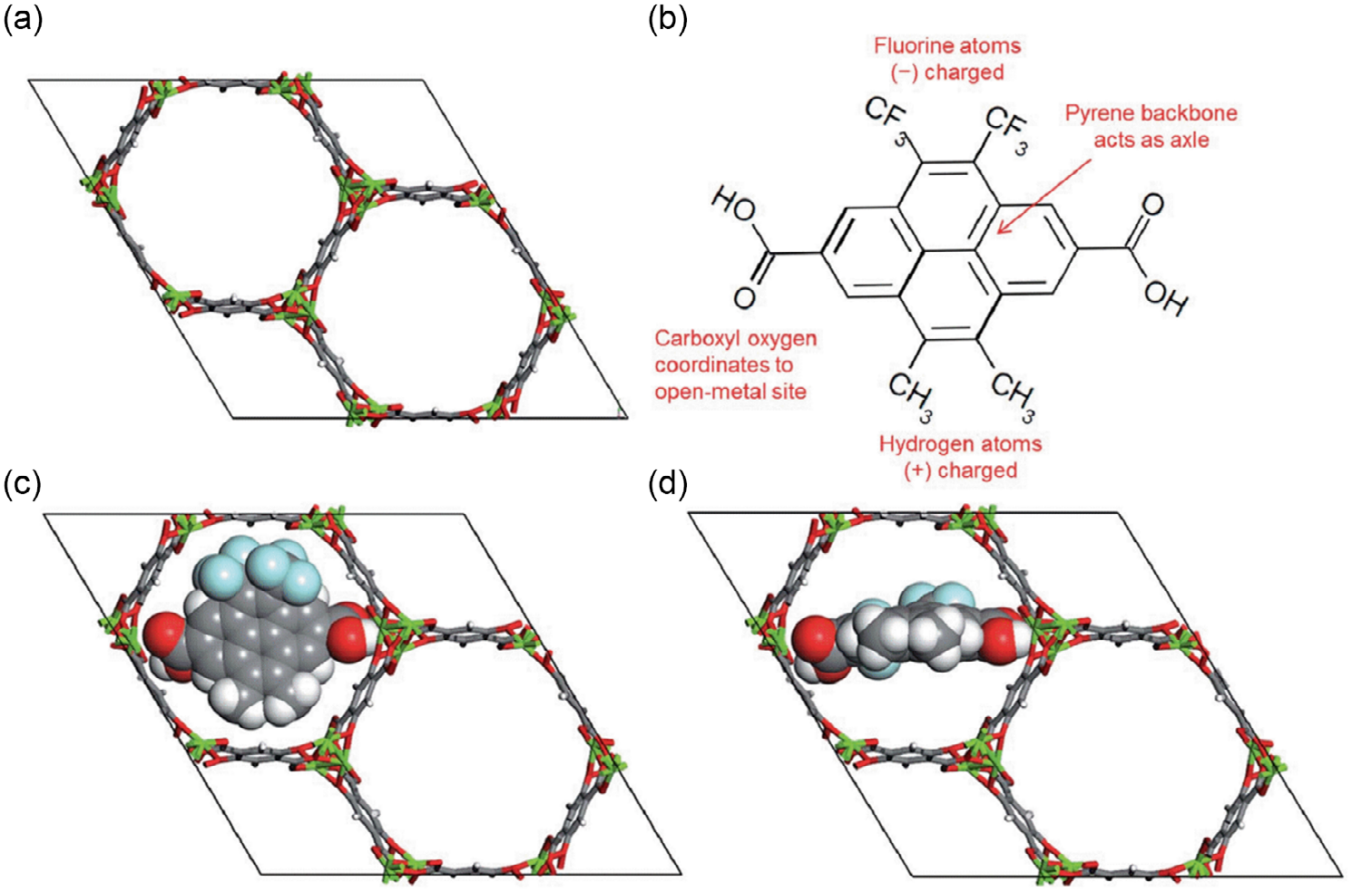
a) Mg–MOF‐74 unit cell, b) chemical structure of the gate molecule, 4,5‐dimethyl‐9,10‐bis(trifloromethyl)pyrene‐2,7‐dicarboxylic acid, c) DFT‐optimized closed gate configuration, and d) open‐gate configuration. Reproduced with permission.^[^ ^76^ ^]^ Copyright 2017, Royal Society of Chemistry.

The regularity and porosity of MOFs make them an ideal platform for molecular machines, with their void space allowing for the movement of MOF building blocks, such as rotating linkers. The conceptual design of E‐field‐regulated molecular gates has been computationally demonstrated by an MD study.^[^ ^76^ ^]^ In this study, the MOF itself does not respond to the E‐field, but it rather acts as a carrier for molecular gates. In the design, a molecular gate was mounted on the open‐metal coordination site within the hexagonal channels of Mg–MOF‐74 (**Figure** **6**a,b). The MOF‐molecular gate complex can be switched between two stable configurations—open and closed—by toggling an external E‐field on and off, as displayed in Figure 6c,d. The switching mechanisms of the gating molecule were elucidated through DFT calculations and MD simulations. The gating molecule containing a permanent dipole moment anchors itself onto the framework and interacts with the E‐field by rotating around its backbone.

### 2.3 COFs

Since the discovery of COFs in 2005, they have become a prominent class of materials and have attracted increasing interest due to their high crystallinity, high stability, and uniformed porosity. Composed of light elements (i.e., H, B, C, N, and O), COFs are interconnected through strong covalent bonds, rendering them comparatively stable. Unlike zeolites and MOFs, the effect of E‐field on COFs has been rarely explored.

Feyter and co‐workers reported E‐field‐mediated reversible transformations between supramolecular networks and COFs.^[^ ^45^, ^77^ ^]^ By using an oriented E‐field in a scanning tunneling microscope, the phase transition between self‐assembled molecular networks and COFs can be locally controlled. The oriented external E‐field facilitated bond formation and bond cleavage reactions. During the phase transformation process, the E‐field‐induced adsorption/desorption of the solvent molecules at negative/positive bias was clearly observed inside the cavity. This revealed the crucial role of solvent molecules in promoting the dynamic network switching process. Notably, the structural transitions triggered by changing E‐field polarity are completely reversible.

According to a research on B_6_N_6_ COF for sensing and delivering anticancer drugs, increasing the negative E‐field strength led to a decrease in the adsorption energy and an increase in the B—O bond length.^[^ ^78^ ^]^ The external E‐field of −0.007 eV Å^−1^ e^−1^ strength changed the adsorption energy from −0.574 to −0.267 eV, and the B—O bond length from 2.949 to 3.021 Å, respectively. The inverse relationship between E‐field strength and adsorption energy was revealed, and thus applying an appropriate E‐field facilitated the drug release from the surface of the B_6_N_6_ COF.

## 3 Applications of CPMs Under E‐Fields

### 3.1 Selective Molecular Adsorption

#### 3.1.1 Molecular Adsorption

It is found that the gas adsorption capacity of polar gas‐adsorbent‐Y zeolites can be increased by applying E‐field, which enhances gas affinity without altering the structure. The SO_2_ adsorption capacity of two Y zeolites (silica and NaY) under an external E‐field was assessed using MD simulations.^[^ ^49^ ^]^ Specifically, an applied external E‐field along the *z* axis resulted in an increased SO_2_ adsorption by ≈12% in both Y zeolites at room temperature, with the adsorbent being completely regenerable. The radial distribution function results illustrated that the application of an external field in both Y zeolites improved the correlation with SO_2_, primarily influencing the changes in adsorption energy, especially enhanced interaction between SO_2_ and Na^+^ ions in NaY zeolites. Moreover, the E‐field increased the ordered orientation of SO_2_ molecules inside the Y zeolites, which creates additional adsorption space, contributing to the enhanced adsorption capacity. Furthermore, the comparative analysis of the two Y zeolites demonstrated that decreasing the Si/Al ratio of the Y zeolite favored the adsorption capacity toward SO_2_. This study demonstrates that E‐fields have the potential to increase the adsorption capacity and adsorption energy of some polar gas adsorbates at computational level.

The hydrogen storage capacity of MOF/carbon (MOFAC) composites can be significantly enhanced by applying an external E‐field.^[^ ^56^ ^]^ The hydrogen adsorption capability of MOFAC3 (MOFAC with 22.2 wt% of carbon) was the highest under an E‐field, showing an increase of about 31.5% compared to MOFAC3 without an external E‐field. The enhanced capacity is attributed to the stronger electrostatic interaction between hydrogen and IRMOF‐8 caused by field polarization. The E‐field induces conductive MOFAC materials, causing IRMOF‐8 to receive charges from adjacent carbon particles, forming localized charge polarization. Then the induced strong electrostatic force can polarize hydrogen molecules, forming enhanced bonds with hydrogen and thus enhancing storage absorption. Moreover, the enhancement by the E‐field showed full reversibility from the adsorption isotherm cycle of the MOFAC3. The low electrical resistance of MOFAC3 gives it conductive properties, thereby making it easily induced by E‐fields. This sets it apart from other traditional CPMs. In addition, this study did not find the optimum condition. Further improving the conductivity of the composite material and thus enhancing the polarization effect is a future research direction.

**Figure 7:**
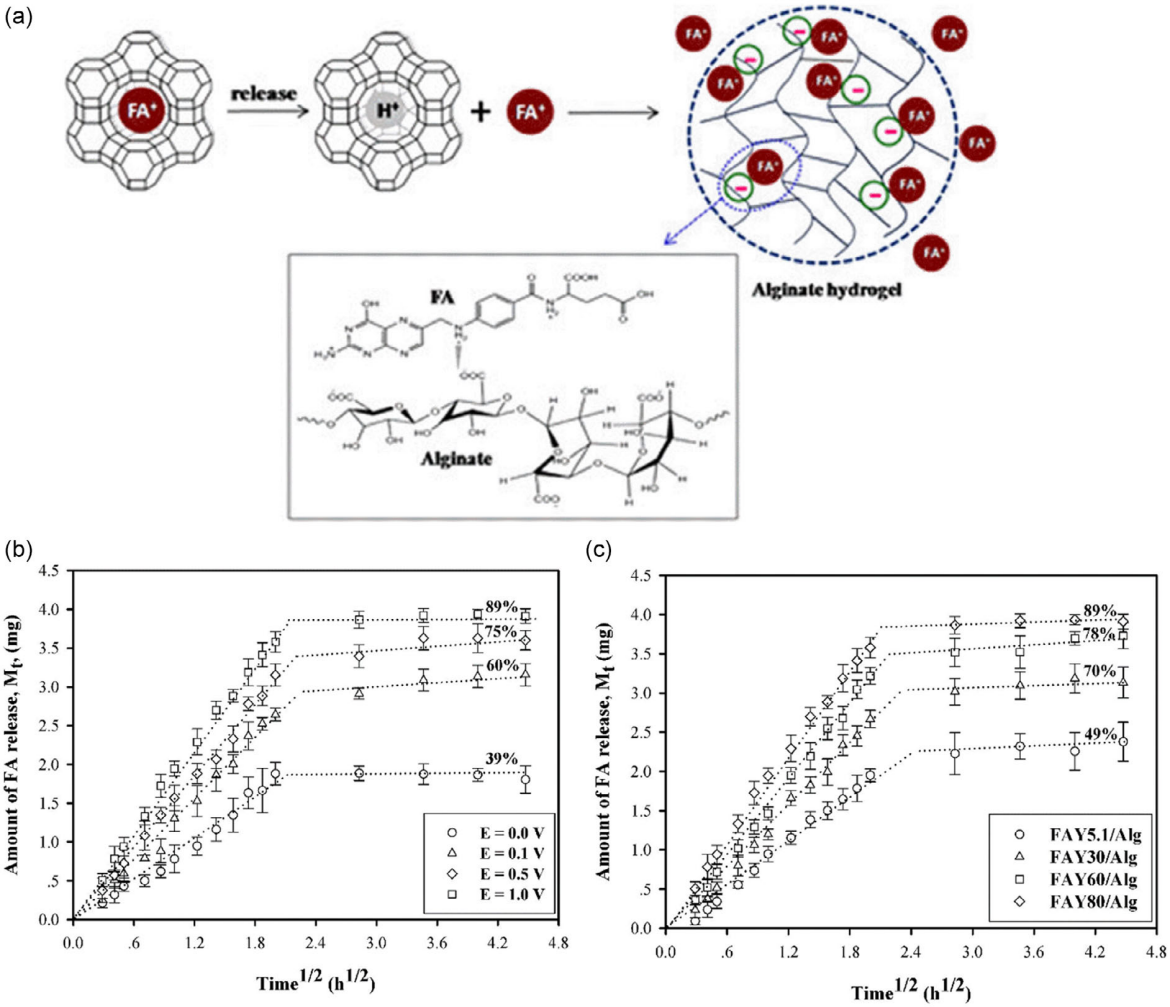
a) Proposed mechanism of FA released from zeolite FAY/alginate hydrogel. b) Amounts of folic acid released from FAY80/Alg_0.7 hydrogels at various electrical potentials. c) Amounts of folic acid released from FAY/Alg_0.7 hydrogels of various Si/Al ratios at *E* = 1 V. Reproduced with permission. Reproduced with permission.^[^ ^79^ ^]^ Copyright 2016, American Chemical Society.

CPMs can store and release specific molecules under the regulation of an E‐field. The release of folic acid (FA) from zeolite Y through ion exchange under E‐field introduces an innovative method for controlled and improved drug delivery.^[^ ^79^ ^]^ Zeolite Y, characterized by its 3D framework capable of encapsulating molecules such as FA, was integrated into an alginate hydrogel to form a zeolite Y/alginate (FAY/Alg) hydrogel, as shown in **Figure** **7**a. Upon application of an E‐field, the positively charged FA molecules are drawn toward the negatively charged structure of the zeolite. Concurrently, an ion‐exchange occurs, allowing FA within the zeolite to swap places with protons from the surrounding buffer, thus balancing the charge of zeolite. The presence of an E‐field, especially with the anode in proximity to the hydrogel matrix, markedly boosts the migration of FA out of the zeolite FAY/Alg hydrogel. This process benefits from the electro‐repulsive forces generated between the positively charged electrode and FA molecules. Notably, an increase in the E‐field strength correlates with a rise in the diffusion coefficient of FA from the hydrogel, enabling a swifter release of the drug, depicted as Figure 7b. Furthermore, the investigation highlighted that a higher Si/Al ratio in the zeolite enhances its drug delivery performance, attributed to the diminished interaction between Al and FA and larger diffusion coefficient for FA, promoting more efficient drug diffusion. This study demonstrates that zeolite/hydrogel is a potential drug matrix that can regulate the timing of release through an E‐field. However, further research on more different drugs is still needed to prove the universality of this method in future drug delivery.

E‐field produced by microwaves can dehydrate zeolites. Microwaves intrinsically generate an E‐field as a constituent component of their propagation, being electromagnetic waves characterized by perpendicularly oscillating E‐field and magnetic field relative to their direction of propagation. In a conducted study on 13X zeolite dehydration by microwaves, it was demonstrated that water molecules were desorbed directly by interacting with the E‐field.^[^ ^80^ ^]^ The proposed microwave dehydration model highlights that zeolite heating does not occur. The desorption rate exhibited a first‐order kinetic relationship, with the rate coefficient inversely proportional to the square of the applied E‐field intensity. This study provides a preliminary explanation for the H_2_O desorption. Further research conducted which can achieve a stable adsorption–desorption cycle will help further develop zeolite‐based water storage materials.

The modulation of MOFs under external E‐field is also applied to the storage and release of drugs using the nanoporous structure. One 2D structure MOF, metal‐organic nanosheet (MON), featured with nanoscale thickness and microscale lateral dimensions, has been used to design a new drug delivery system.^[^ ^81^ ^]^ Gemcitabine conjugated to polypyrrole (PPy) polymer was loaded on the copper benzene dicarboxylate (CuBDC) Hong Kong University of Science and Technology MON. DFT calculations indicated that covalent bonding between the drug and polymer negligibly affected the chemical properties of the drug. The MD simulation suggested a higher magnitude of the external E‐field would increase the adsorptive interactions between the gemcitabine–PPy complex and the CuBDC substrate, facilitating the better adsorption of the complex on the substrate. Conversely, a lower E‐field intensity facilitated the release of the drug at the drug target. This designed MON has theoretically showed the possible E‐field response effects of such materials. However, there is a lack of in‐depth explanation of the E‐field‐induced mechanism. Successfully synthesizing corresponding materials is challenging and there is still a long way to go to achieve practical application.

COFs can be specially designed to store and release particular molecules under E‐fields. The primary effect of the E‐field on the molecular adsorption of porous COFs is the reduction of adsorption energy between the adsorbed molecules and the COF. For example, COF can be used in the release of anticancer drugs^[^ ^78^ ^]^ and the removal of organic pollutants phenol from industrial wastewater.^[^ ^82^ ^]^ The capability of B_6_N_6_ COF as a desired carrier for sensing and delivering anticancer drugs, such as lomustine, was investigated using DFT calculations.^[^ ^78^ ^]^ The computational results indicated that a negatively increased external E‐field decreased the adsorption energy and increased the B—O bond length. Therefore, the presence of a negative external E‐field along the z axis facilitated the release of lomustine drug from the B_6_N_6_ nanosheet. Another study explored the adsorption behaviors of phenol in industrial wastewater onto the non‐hydroxyl COFs^[^ ^83^ ^]^ under varying E‐field strengths, using MD and well‐tempered metadynamics simulations.^[^ ^82^ ^]^ The findings reveal that an increased E‐field reduces the phenol molecular adsorption on the COF. A notable decrease in adsorption energy with a 1 V·nm^−1^ applied E‐field was due to the enhanced rigidity from π − π interactions within the benzene ring of phenol. Additionally, higher E‐fields reduce phenol diffusion on COF surfaces and channels. This is attributed to the strengthened hydrogen bonds between the hydroxyl groups of phenol and water molecules, which diminish adsorbate–carrier interactions. Therefore, E‐fields can serve as stimuli for controlled and drug delivery an phenol removal using COFs, highlighting their potential. However, current COFs being utilized for molecular storage and release under E‐field are quite limited and can only work for specific molecules, which indicates that there is a considerable gap before practical application.

#### 3.1.2 Selective Molecular Adsorption for Separation

**Figure 8:**
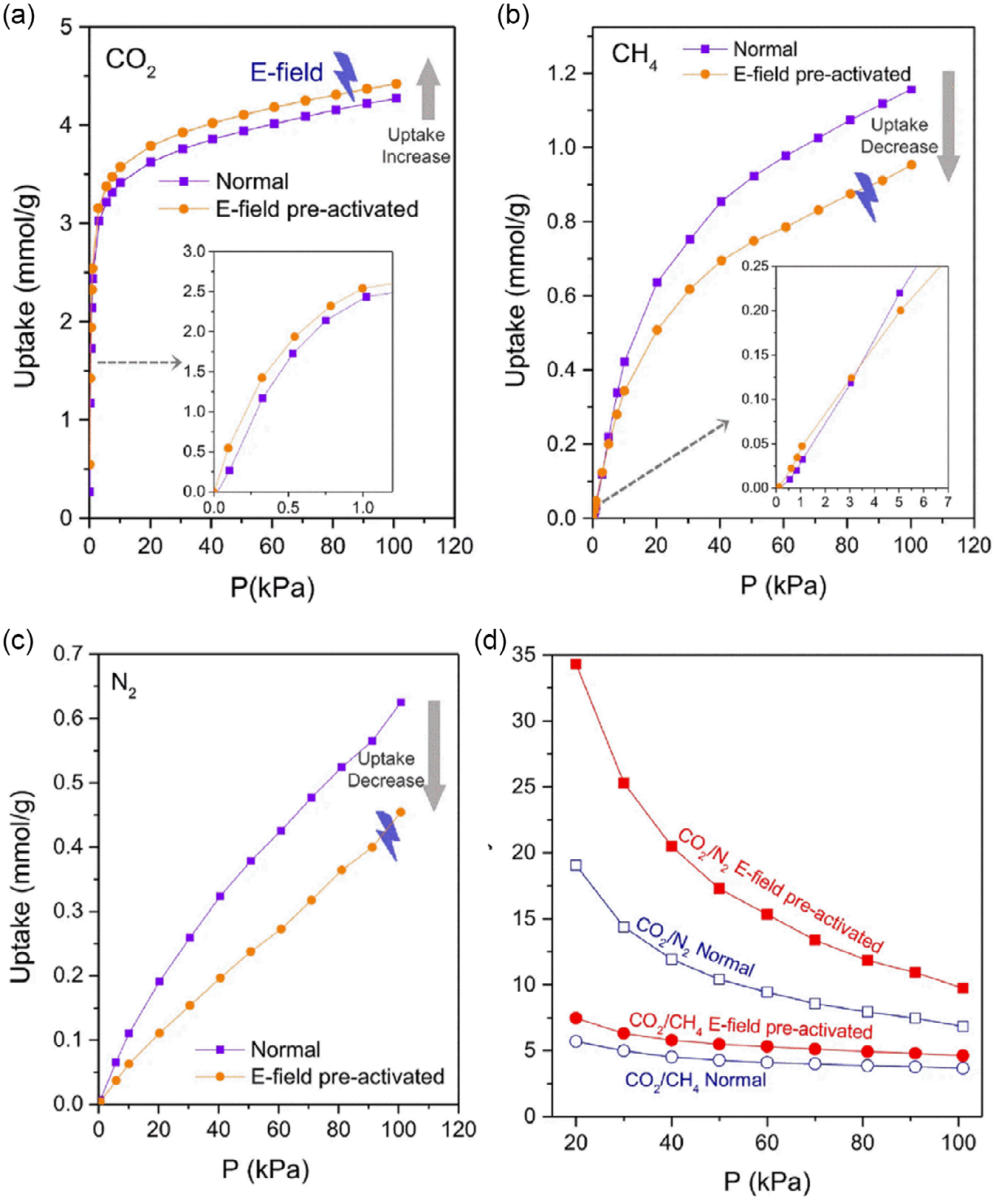
Effect of E‐field pre‐activation on gas adsorption in r2KCHA zeolites. Adsorption isotherms of a) CO_2_, b) CH_4_, and c) N_2_ on r2KCHA at 273 K without and with the E‐field pre‐activation. d) Selectivity of CO_2_/CH_4_ and CO_2_/N_2_ on r2KCHA were increased by the E‐field pre‐activation. Reproduced with permission.^[^ ^37^ ^]^ Copyright 2023, Springer Nature.

The gas adsorption selectivity in zeolite molecular sieves can be modulated by an external E‐field. Pre‐activation with an E‐field during the degas process enhanced CO_2_ adsorption and reduced CH_4_ and N_2_ adsorption in r2KCHA,^[^ ^37^ ^]^ as shown in **Figure** **8a** **–**c. The changed adsorption performance led to over 25% improvement in CO_2_/CH_4_ and CO_2_/N_2_ separation selectivity (Figure 8d). Importantly, the enhanced separation performance of E‐field pre‐activated zeolites was sustained across multiple adsorption/desorption cycles. The pre‐activated zeolite exhibited cation relocation and framework expansion, with the vacant cation sites serving as new adsorption sites for CO_2_ molecules, thereby increasing the CO_2_ adsorption capacity. Furthermore, framework expansion and decreased cation–guest interaction caused reduced adsorption energy for CH_4_ and N_2_, thus decreasing their adsorption uptakes. The E‐field pre‐activation process significantly enhanced the adsorption selectivity of CO_2_ over CH_4_ and N_2_. This strategy was also applied to other zeolites, such as ZSM‐25 and TMA‐Y. It is noteworthy that cation relocation caused by E‐field also facilitated a “gate‐opening” effect in trapdoor zeolites, allowing the admission of gas molecules blocked by the trapdoor ions. This study revealed the E‐field‐induced structural transformation of zeolites, which has guiding significance for the future use of E‐fields to regulate the participation of zeolites in gas separation.

External E‐fields have also been reported to influence the permeance of both pure (N_2_, O_2_, CO_2_) and binary systems (CO_2_/N_2_ and O_2_/N_2_ gas mixtures) of mordenite framework inverted (MFI) zeolite membrane.^[^ ^53^ ^]^ The gas separation performance was evaluated using MD simulations operated at different frequencies and strengths of E‐fields, normal to the membrane surface. Results indicated that in pure systems, while the permeance of N_2_ and O_2_ remained unaffected by E‐fields, the permeance of CO_2_ slightly decreased. For gas mixtures (CO_2_/N_2_ and O_2_/N_2_), the presence of E‐fields led to decreased permeance for both gases. For CO_2_/N_2_ mixtures, a more significant reduction for CO_2_ was observed, resulting in a lower separation factor. However, for O_2_/N_2_ mixtures, the separation factor remained essentially unchanged. Additionally, observations on mean‐square displacement (MSD) and membrane adsorption suggested that applying E‐fields reduced adsorption but increased the diffusion rate. Overall, external E‐fields did not improve the separation efficiency significantly for the MFI membrane. The reasons for these phenomena remain unclear, potentially related to changes in the active sites and adsorption energy within MFI. Further theoretical and experimental research is required for verification.

**Figure 9:**
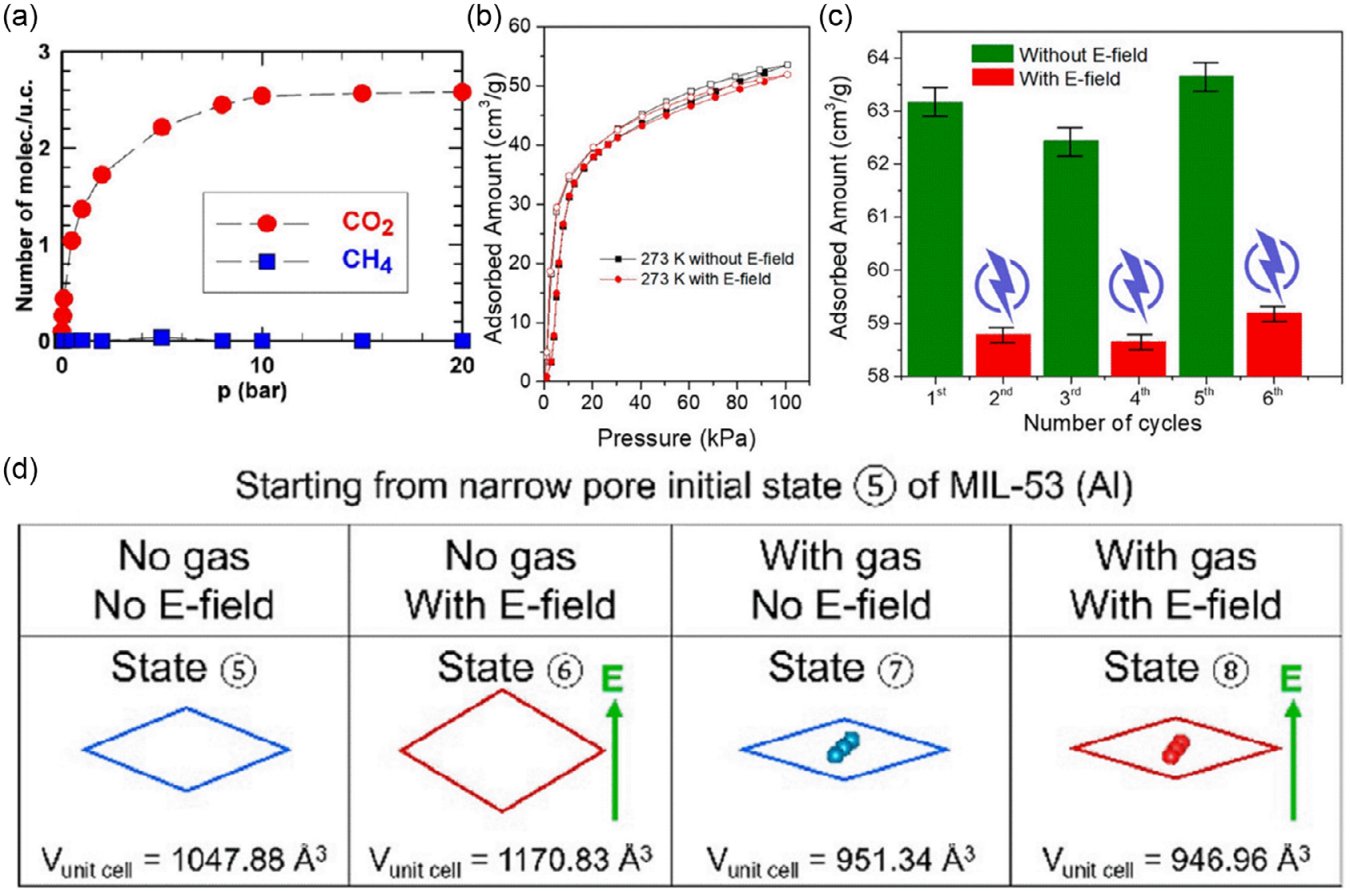
a) Simulated adsorption isotherm of CO_2_ (circle) and CH_4_ (square) of 50:50 CO_2_/CH_4_ mixture at 300 K under an E‐field of 2.0 V nm^−1^. Reproduced with permission.^[^ ^57^ ^]^ Copyright 2017, American Chemical Society. b) CO_2_ adsorption and desorption isotherms in the synthesized MIL‐53(Al) with and without an E‐field for temperatures at 273 K. c) Cyclic CO_2_ adsorption capacity at 100 kPa and 252 K with the E‐field switched on and off alternatingly. d) Structures of MIL‐53(Al) in the presence/absence of CO_2_ and/or E‐field starting from a narrow pore initial state. Reproduced with permission. Reproduced with permission.^[^ ^84^ ^]^ Copyright 2022, American Chemical Society.

The structural contraction of MIL‐53(Cr) from an LP to an NP state under an external E‐field can influence its gas selectivity. The external E‐field can help maintain the breathing MOF in the NP form during adsorption.^[^ ^57^ ^]^ This observation demonstrates that an applied external E‐field can induce the structural contraction of both empty and guest‐accommodated MIL‐53(Cr) LP forms. Compared to the empty LP form, the relatively strong interactions between guest molecules and host framework exerting internal pressure results in a more continuous structure transition. The CO_2_/CH_4_ mixture adsorption isotherm for MIL‐53(Cr) was given by hybrid osmotic Monte Carlo simulations, predicting a negligible adsorption of CH_4_ and a substantial adsorption of CO_2_, as illustrated in **Figure** **9**a. The highly selective separation of CO_2_ over CH_4_ via a steric effect is attributed to the MIL‐53(Cr) NP form with a 3.5 Å pore size. Therefore, the application of an external E‐field can finely tune the pore size of MIL‐53(Cr), enabling remarkable CO_2_/CH_4_ gas separation performance.

Despite similar structural transitions, changes in the selectivity of MIL‐53(Al) under an external E‐field are influenced by the binding energy of the gas molecule in the host framework. Li et al. demonstrated that the adsorption of small gas molecules in NP MIL‐53(Al) can be regulated by an external E‐field below the gas breakdown voltage.^[^ ^84^ ^]^ The CO_2_ adsorption capacity in NP MIL‐53(Al) at 273 K decreased by ≈7% under a low DC E‐field (Figure 9b). Alternatingly switching the E‐field on and off caused a reversible regulation of CO_2_ adsorption (Figure 9c). DFT calculations revealed that external E‐field induces a structural expansion in the empty NP‐state MIL‐53(Al), while it does not trigger a structural transformation in the presence of CO_2_ molecules (Figure 9d). In contrast to MIL‐53(Cr), the reduced CO_2_ adsorption capacity of NP state MIL‐53(Al) under an E‐field was ascribed to the significantly decreased binding energy rather than framework transformation. This reduction is due to the decreased charge redistribution of the adsorbed CO_2_ molecule and the bridging μOH group within the framework. The study of MIL materials shows that MOFs with similar structures can also have very different changes. The next research direction is to find the optimal strength of structural transformation.

In another example, gas permeation performance through the ZIF‐8 membrane could be regulated in situ by applying an external E‐field, causing the MOF partially transform into monoclinic and triclinic polymorphs with more stiffened lattices.^[^ ^33^ ^]^ The separation factor for the C_3_H_6_/C_3_H_8_ mixture increased from 6 to 8 after applying the E‐field. In comparison, the E‐field had no effect on the permeability of Hong Kong University of Science and Technology (HKUST)‐1 membrane. The stiffened lattice, with reduced rotational freedom and molecular sieving due to changed pore diameter, is considered the cause of switching the gas transport through the ZIF‐8 layer. ≈30% of the ZIF‐8 membrane layer went through polymorphic transformation, leading to a significant change in separation selectivity for gas molecules. Therefore, higher E‐fields suggest the potential for achieving a complete phase transformation, further enhancing gas separation performance.

The binding energy of *M*–MOF‐74 (M = Mg, Mn, Fe, Co, Ni, Cu, Zn) system is also affected by the external E‐field and thus modulates their selective adsorption behavior of small gas molecules.^[^ ^55^ ^]^ In this theoretical study, the external E‐field preferentially strengthens the π* back‐bonding between the transition metal sites and N_2_ molecule, while impairing the σ bond with other gases like CO_2_ and CH_4_. Notably, simulation results suggest that Co–MOF‐74 and Fe–MOF‐74 exhibit a twofold enhancement in N_2_ binding energy compared to CH_4_ under an E‐field, suggesting a potential strategy for improving N_2_/CH_4_ separation selectivity by applying E‐field to modulate binding energy difference. This study explains the energy changes between transition metal sites and gas molecules from the perspective of bonding under E‐field, which helps to deepen our understanding of the MOF adsorption process under E‐field.

The concept of E‐field‐controlled molecular gates mounted on MOFs showed a possible way to control the flow of methane molecules in the channels of Mg–MOF‐74. The conceptional Mg–MOF‐74 system was tested with CH_4_ molecules placed between the molecular gates and a graphene wall by MD simulation.^[^ ^76^ ^]^ The simulation results show that methane molecules can be blocked or allowed to diffuse through MOF under the control of molecular gates which is similar to nanoscale butterfly valves (Figure 6c,d). The gate molecule has a carboxyl group to coordinate with the open‐metal site and binds tightly to the MOF framework. It also has a permanent dipole to respond to the E‐field. Without applying an E‐field for 30 ns, the gate remains in a closed configuration despite the pressure exerted by the methane molecules, and the methane molecule cannot pass through. The E‐field of 3 V nm^−1^ was then turned on and held for 30 ns; as the E‐field was applied, the molecular gate rotated and switched to the open configuration, with methane molecules diffusing into the empty channels. Different from the response of CPMs to E‐field, the gas diffusion controlled by valves provides a new idea for gas separation. This valves‐controlled CH_4_ diffusion provides a new idea for gas separation using CPM as a confinement framework and E‐field‐responsive molecules as gates.

**Figure 10:**
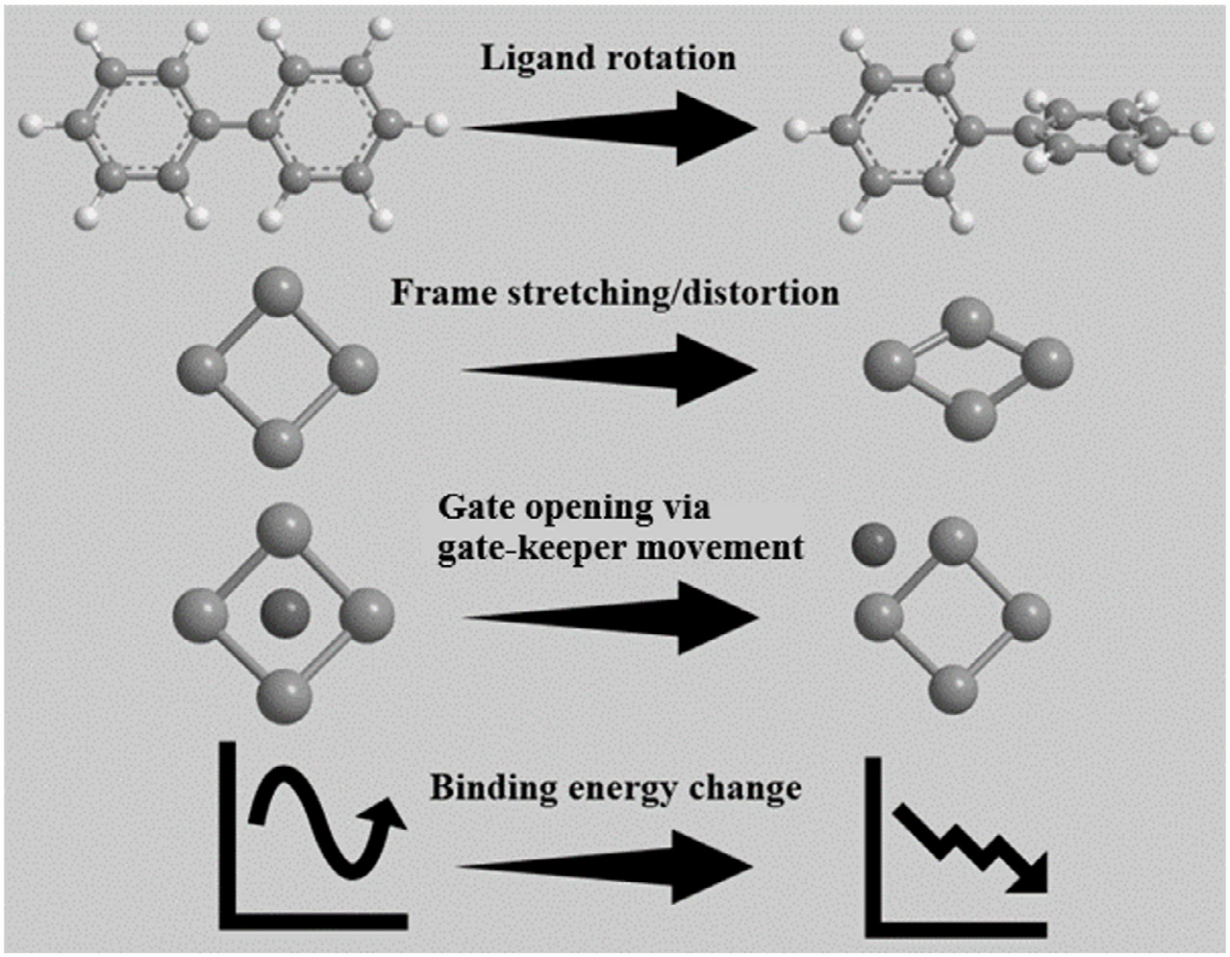
Possible phenomena of CPMs induced by electric field in gas adsorption.

So far, we have discussed the molecular adsorption behavior of CPMs under E‐field. Regarding the behavior of CPMs with specific adsorbed gas molecules under E‐field, we summarized the possible behaviors of CPMs reported in **Figure** **10**.

### 3.2 Selective Ion Adsorption

#### 3.2.1 Ion Adsorption

**Figure 11:**
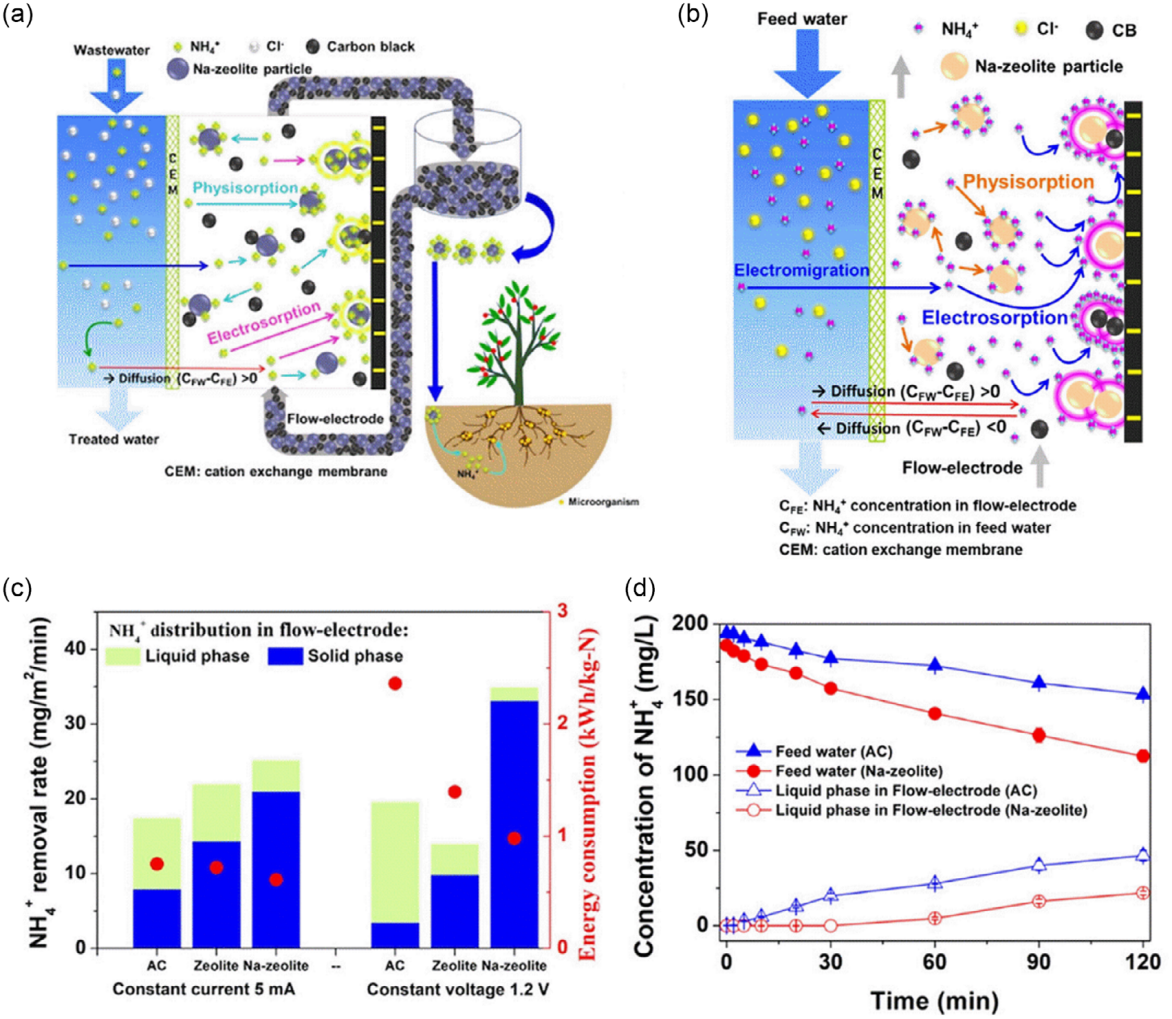
a) Flowchart of a novel Na–zeolite‐based FCDI process for removing NH^4+^ and recovering fertilizer resources. b) Illustration of the ion transport and removal processes, including electromigration, diffusion, electrosorption, and adsorption. c) NH^4+^ recovery via FCDI under CV and CC conditions. d) NH^4+^ removal and recovery from the food waste fermentation liquor using FCDI. Reproduced with permission. Reproduced with permission.^[^ ^71^ ^]^ Copyright 2023, American Chemical Society.

The zeolite framework contains exchangeable alkali or alkaline‐earth metal cations (typically Na, K, Ca, and Mg), which can be reversibly replaced by surrounding cations. The ion adsorption capability of zeolite can be improved through enhancing the ion‐transport rate. He et al. have developed a low‐cost, Na‐enhanced Na–zeolite and carbon black composite flow‐electrode (FE) suspension for the removal and recovery of NH_4_^+^ using flow‐electrode capacitive deionization (FCDI) technology.^[^ ^71^ ^]^ As shown in **Figure** **11a** **,**b, the electrically charged carbon black particles electrosorb a limited quantity of NH_4_^+^ and facilitate charge transfer from the current collector to the Na–zeolite particles. Additionally, NH_4_^+^ ions released from uncharged carbon black particles get adsorbed by the Na–zeolite within the suspension. These charge transfers are vital for enhancing NH_4_^+^ electrosorption by the Na–zeolite. NH_4_^+^ ions adsorbed onto the surface of Na–zeolite are progressively swapped with other ions in the inner pores of the Na–zeolite particles, significantly improving the overall NH_4_^+^ elimination and recovery efficiency of FCDI system. As demonstrated in Figure 11c, the Na–zeolite exhibits a heightened affinity for NH_4_^+^ compared to traditional activated carbon and zeolite, leading to a substantial increase in NH_4_^+^ adsorption capacity, with respective growth rates of 44% and 14%. This enhancement permits the Na–zeolite to efficiently extract NH_4_^+^ ions from the feed water and accumulate them within the FE suspension, thereby aiding in the reduction of NH_4_^+^ levels in the FE suspension and reducing the effects of back diffusion for better NH_4_^+^ elimination. This research provides a perspective to deal with eutrophic or polluted water in the future.

**Figure 12:**
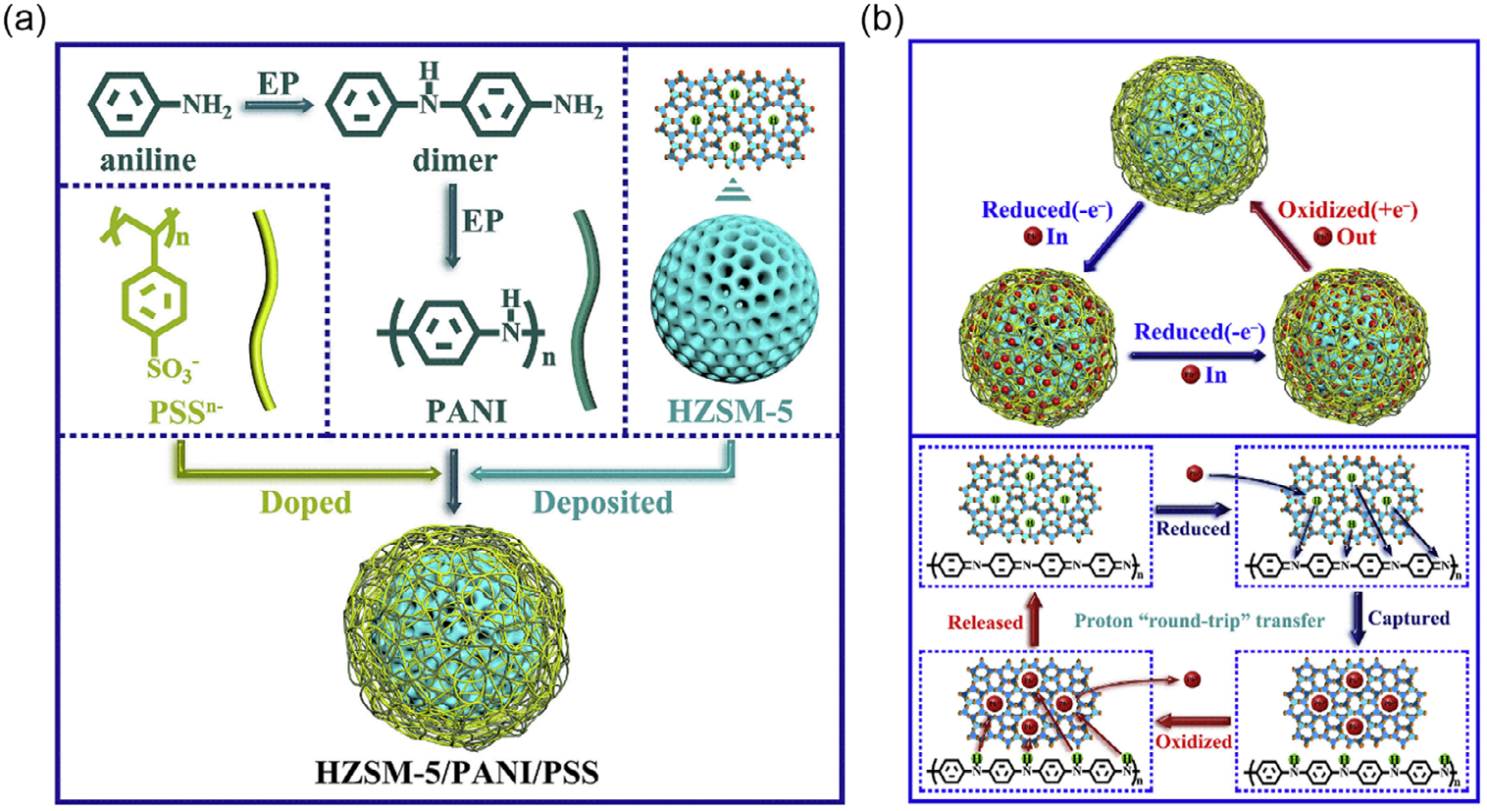
a) The preparation steps and composition of HZSM‐5@PANI/PSS‐based film. b) Schematic illustration of the electrochemically switched ion‐exchange process. Reproduced with permission. Reproduced with permission.^[^ ^85^ ^]^ Copyright 2018, Elsevier.

Du et al. explored the creation and use of an innovative porous electroactive composite film constructed from HZSM‐5@PANI/PSS core–shell nanospheres, aiming for the swift and selective adsorption and desorption of Pb^2+^ ions in water solutions.^[^ ^85^ ^]^ HZSM‐5 zeolites were connected with polyaniline/polystyrene (PANI/PSS) polymers to boost electron movement. Upon electrochemical reduction at a designated potential, the composite film initially attracts Pb^2+^ ions to the PANI/PSS exterior due to the necessity for charge neutrality. Then, at a reduced potential, these ions move to the HZSM‐5 center, getting captured by the oxygen atoms of zeolite framework, thereby efficiently capturing Pb^2+^ ions. Conversely, reoxidizing the reduced composite film at a different potential causes the Pb^2+^ ions to be released back into the aqueous solution. This reversible process of Pb^2+^ ion absorption and release are modulated by altering the redox state of the composite film via electrical voltage (**Figure** **12**). Unlike ion exchange, as shown in the previous example, this method of selective ion separation benefits from the confining effect of zeolite straight channels, which have precise pore sizes facilitating easier dehydration of Pb^2+^ ions over other heavy metals due to the lower Gibbs energy required for Pb^2+^ dehydration, leading to their preferential absorption by HZSM‐5. Overall, the novel “round‐trip” proton‐transfer mechanism, driven by the electrochemical properties of HZSM‐5 zeolite and film, allows for the reversible capture and release of Pb^2+^ ions.

**Figure 13:**
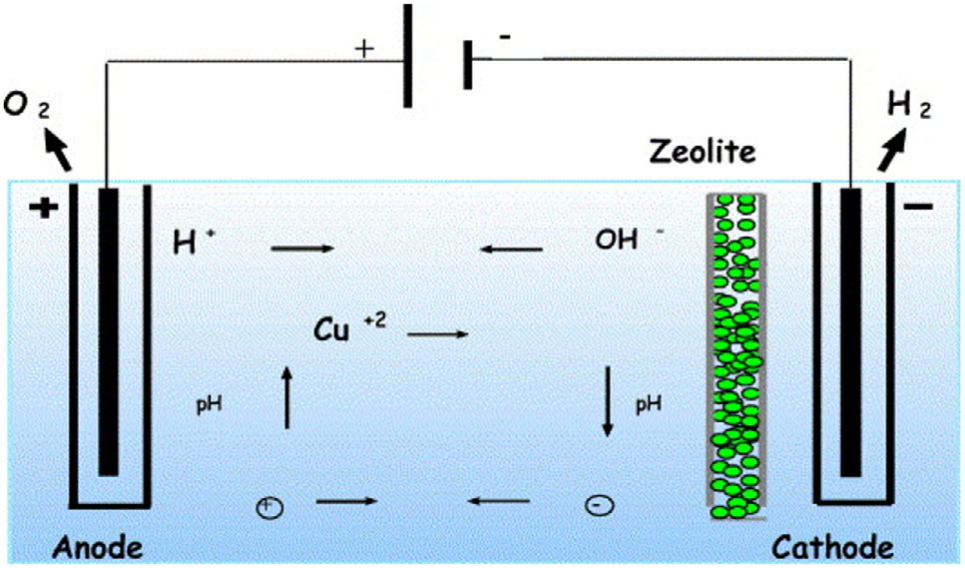
A schematic picture of ions electric field migration toward the electrodes and the zeolite slice to trap the Cu^2+^ ions. Reproduced with permission. Reproduced with permission.^[^ ^86^ ^]^ Copyright 2006, Elsevier.

In the process of electrokinetic soil decontamination, E‐field‐induced zeolite 13X outperforms traditional liquid‐based approaches in capturing heavy metals.^[^ ^86^ ^]^ This advantage is tied to cation‐exchange capacity (CEC) of zeolite 13X, a feature that enables it to seize cations, such as Cu^2+^, by swapping them with alternative cations within its own structure. The effectiveness of this mechanism is significantly enhanced under the dynamic and ion‐mobilizing conditions created by the E‐field within the electrokinetic setup. When zeolites are incorporated into the electrokinetic apparatus, they form a unique interface area. This arrangement and the ensuing E‐field expedite the movement of heavy metal ions toward the zeolite, raising the likelihood of their interception and subsequent adsorption by the zeolite structural cavities (**Figure** **13**). The intricate network of channels and cavities within the zeolites serves as efficient traps for these ions, thus averting their accumulation at the cathode. Even with the competitive presence of H^+^ during the electrokinetic process, zeolites demonstrate a remarkable ability to capture and accumulate heavy metal ions moving toward the cathode. In addition, when clay is used in the electrokinetic scenario, CEC performance remains commendable, underscoring its practical superiority in such applications.

The understanding and development of E‐field‐induced ion adsorption based on COFs still require further exploration. One promising application of COFs is the E‐field enhanced adsorption of uranium ions. Zhang et al. have designed and synthesized a 3D sp_2_ carbon‐linked COF, termed TFPM–PDAN–AO (TFPM: tetrakis(4‐formylphenyl)methane, PDAN: 2,2′‐(1,4‐phenylene)diacetonitrile, AO: amidoxime groups), tailored for uranium‐ion adsorption.^[^ ^87^ ^]^ The presence of amidoxime groups on the COF furnishes binding sites for uranyl ions. Upon the application of a negative bias, uranyl ions selectively adhere to these sites on the electrode surface. Consequently, U(VI) can be subject to precipitation as Na_2_O(UO_3_H_2_O)_x_ in the presence of Na^+^ as a charge‐neutral species. Upon bias removal, uranium ions and uranium complexes stay on the electrode surface while other nonspecifically bound ions redistribute and release from surface‐active sites. This mechanism facilitates the efficient electroextraction of uranium from seawater using the 3D COF framework. Similarly, Yang et al. harnessed carboxyl‐functionalized COF‐1 and COF‐2 as cathode and anode materials for the electroadsorption of UO_2_^2+^ and ReO_4_^−^.^[^ ^88^ ^]^ A substantial electric driving force engenders high double‐layer capacitance, fostering the adsorption of U(VI) and Re(VII) on the functionalized electrode surface by mitigating Coulombic repulsion between adsorbed ions and mobile ions. The adsorption rate and capacity were amplified by over twofold in comparison to conventional physical and chemical methods, indicative of high efficiency and rapid kinetics. These studies shed light on the potential of E‐field‐ion adsorption based on COFs, showcasing promising avenues for ion adsorption and extraction processes utilizing tailored COF platforms with functional groups for specific ion binding and electroadsorption mechanisms. However, the small adsorption capacity, high energy consumption, and low efficiency still restrict their applications.

#### 3.2.2 Selective Ion Adsorption for Separation

**Figure 14:**
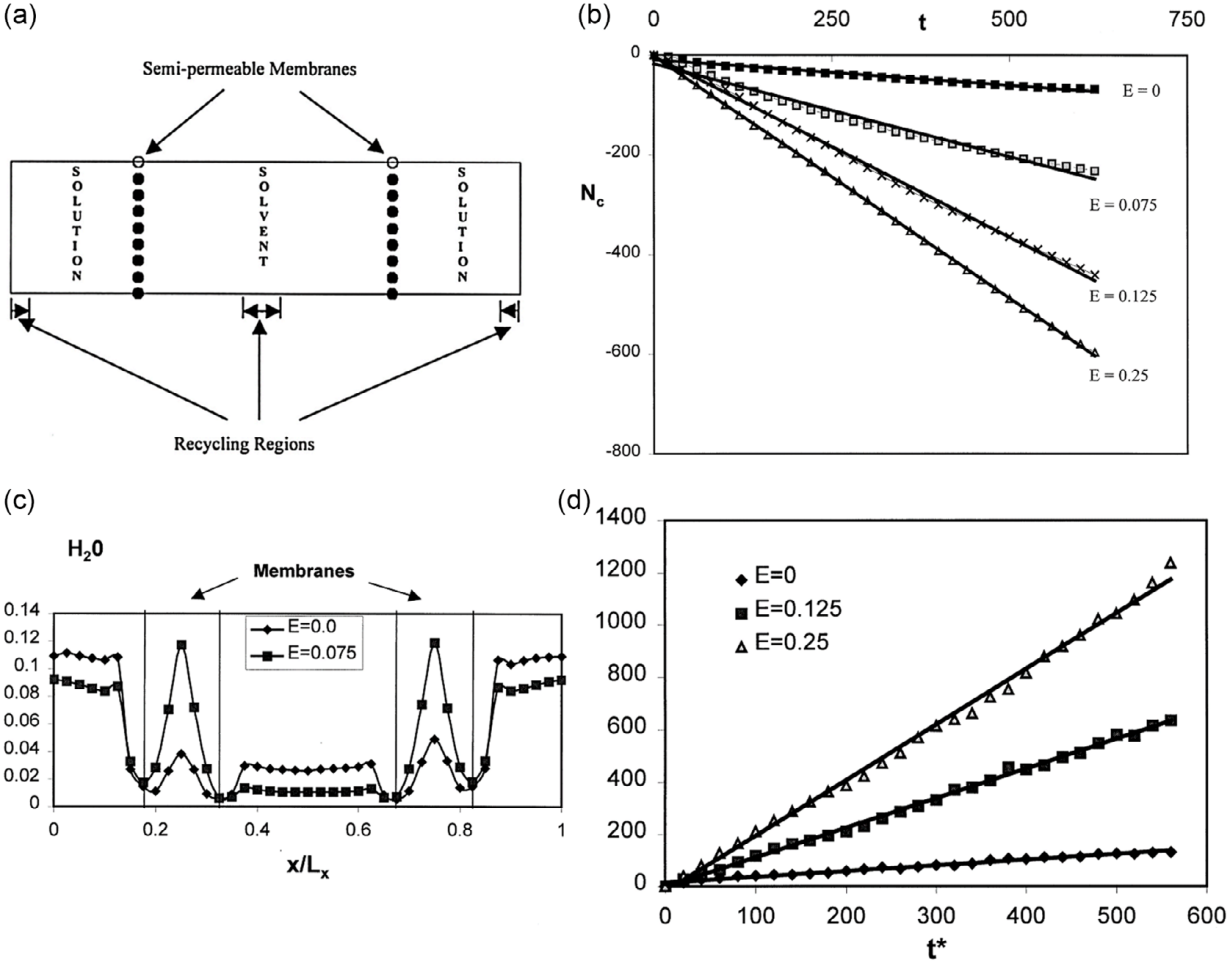
a) Schematic of the simulation system. b) Effect of an external electric field on the number of solvent (water) molecules permeating the membrane as a function of time. c) Density profile of the solvent molecules in the simulation system at steady state for with an electric field. d) Effect of external electric fields on the mean‐squared displacement (MSD) of solvent molecules perpendicular to the membrane plane. Reproduced with permission. Reproduced with permission.^[^ ^89^ ^]^ Copyright 2002, American Chemical Society.

An applied external E‐field plays a significant role in accelerating separation rates of ZK‐4 zeolite membranes in the separation of supercritical aqueous NaCl mixtures.^[^ ^89^ ^]^ This study employs molecular simulations to explore the separation performance, with schematic of the simulation system depicted in **Figure** **14**a. The region between the two ZK‐4 membranes was filled with solvent (water) molecules, and the adjacent exterior regions of both membranes were occupied with solution (NaCl–water mixtures). As Figure 14b demonstrates, the permeation rate of water molecules across the membranes increased up to fifteenfold when the E‐field strength is adjusted from zero to a reduced field of *E* = 0.25 (a reduced field of *E* = 0.1 corresponds to a voltage of ≈465 mV across the membrane). This permeation enhancement is attributed to both increased solvent adsorption within the zeolite pores and a higher diffusion coefficient of water molecules in the presence of elevated E‐field strengths. Specifically, water adsorption within the zeolite cavities triples due to the alignment of water dipoles and the disruption of ion–water and water clusters, which lower the entrance resistance for solvent flux. Concurrently, the diffusion coefficient increase is a consequence of weakened ion–water molecular interactions, facilitating more liberal movement of water. The findings imply that ZK‐4 zeolite membranes could be used for the effective separation in polar supercritical solvents through E‐field regulation. However, it is noted that since the membranes studied were only half to one unit cell thick, they were too thin to directly compare the permeability with experimental values, which also made it more difficult to translate this theory into practical applications.

**Figure 15:**
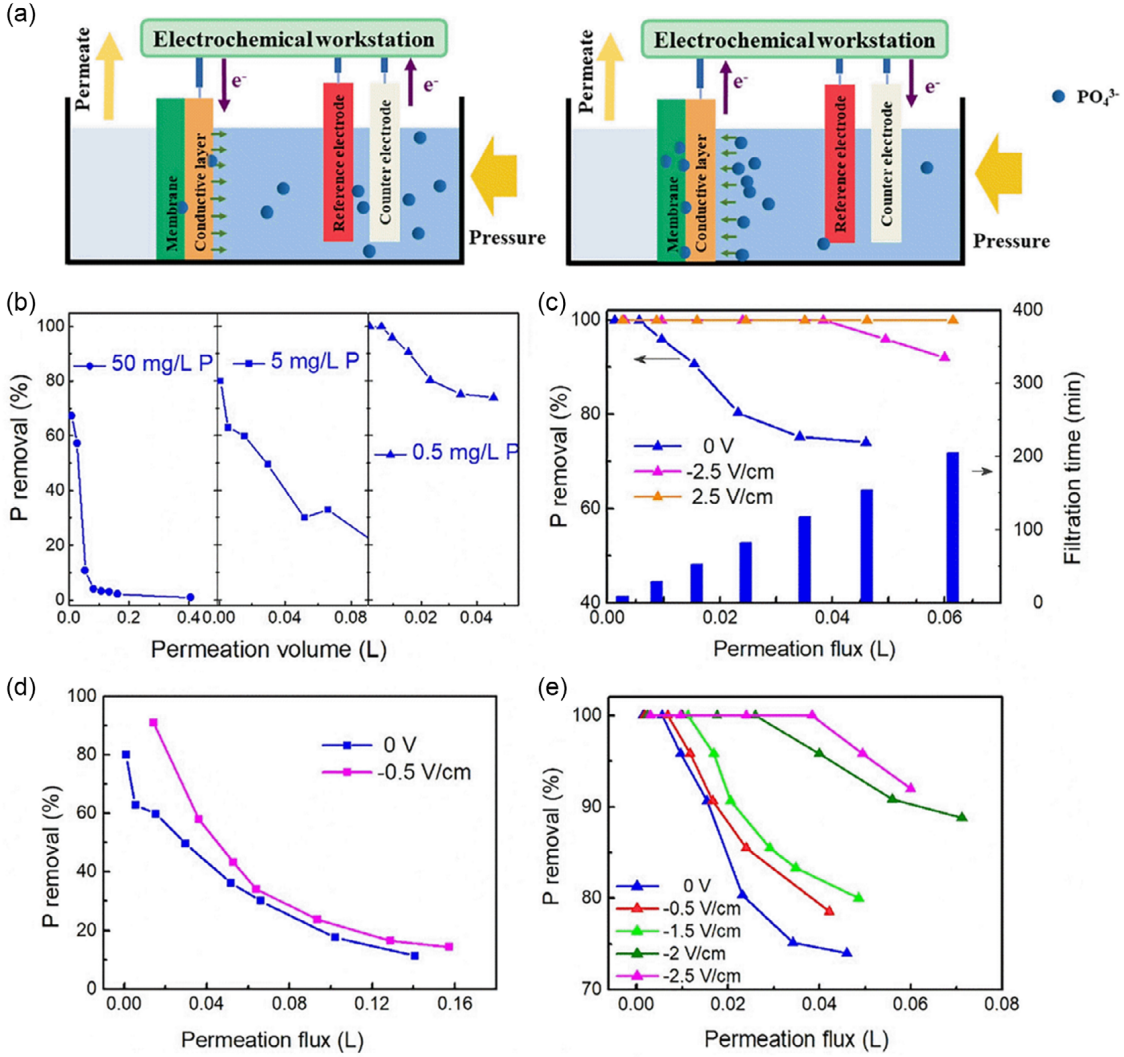
a) The mechanism for electric‐field‐enhanced performance of filtration system. The phosphate rejection of CC/MMM b) with different initial phosphate concentrations in feed solution, c) under different applied electric fields (0.5 mg P L^−1^), d) under different applied electric fields (5 mg P L^−1^), and e) under different applied voltages. Reproduced with permission. Reproduced with permission.^[^ ^90^ ^]^ Copyright 2019, Elsevier.

Liu et al. has developed an innovative filtration method utilizing an MOF‐enhanced ultrafiltration mixed matrix membrane (MMM) equipped with capacitive deionization (CDI) capabilities, aimed at the improved elimination of phosphate ions (**Figure** **15**).^[^ ^90^ ^]^ The inclusion of NH_2_–MIL‐101(Al), known for their functional groups that possess a strong affinity toward phosphate ions, facilitating their selective absorption and entrapment during the filtration process. This study has revealed structural changes in NH_2_–MIL‐101(Al) post‐phosphate adsorption, suggesting a ligand exchange between phosphate ions and hydroxyl groups, alongside electrostatic attraction and the creation of extra adsorption sites courtesy of the amine groups present in the MOFs. The application of an E‐field augments the membrane's efficacy in segregating phosphate ions by directing the anions toward the positively charged surface of the membrane when acts as an anode. This enhancement in the adsorption process leads to a more effective phosphate elimination of NH_2_–MIL‐101(Al), up to 100% (Figure 15c). At an E‐field strength of 1 V cm^−1^, the adsorption capability of CC/MMM surpasses that of CC/PES and outperforms CC/MMM in the absence of an E‐field. Figure 15b,e demonstrates that reduced phosphate concentrations and elevated voltages contribute to better filtration efficiency. What makes this study distinguished from other studies on CPMs for CDI is that the membrane behaves completely reversibly as anode and cathode electrodes, while having extremely high removal rates, which give this membrane great potential for application.

**Figure 16:**
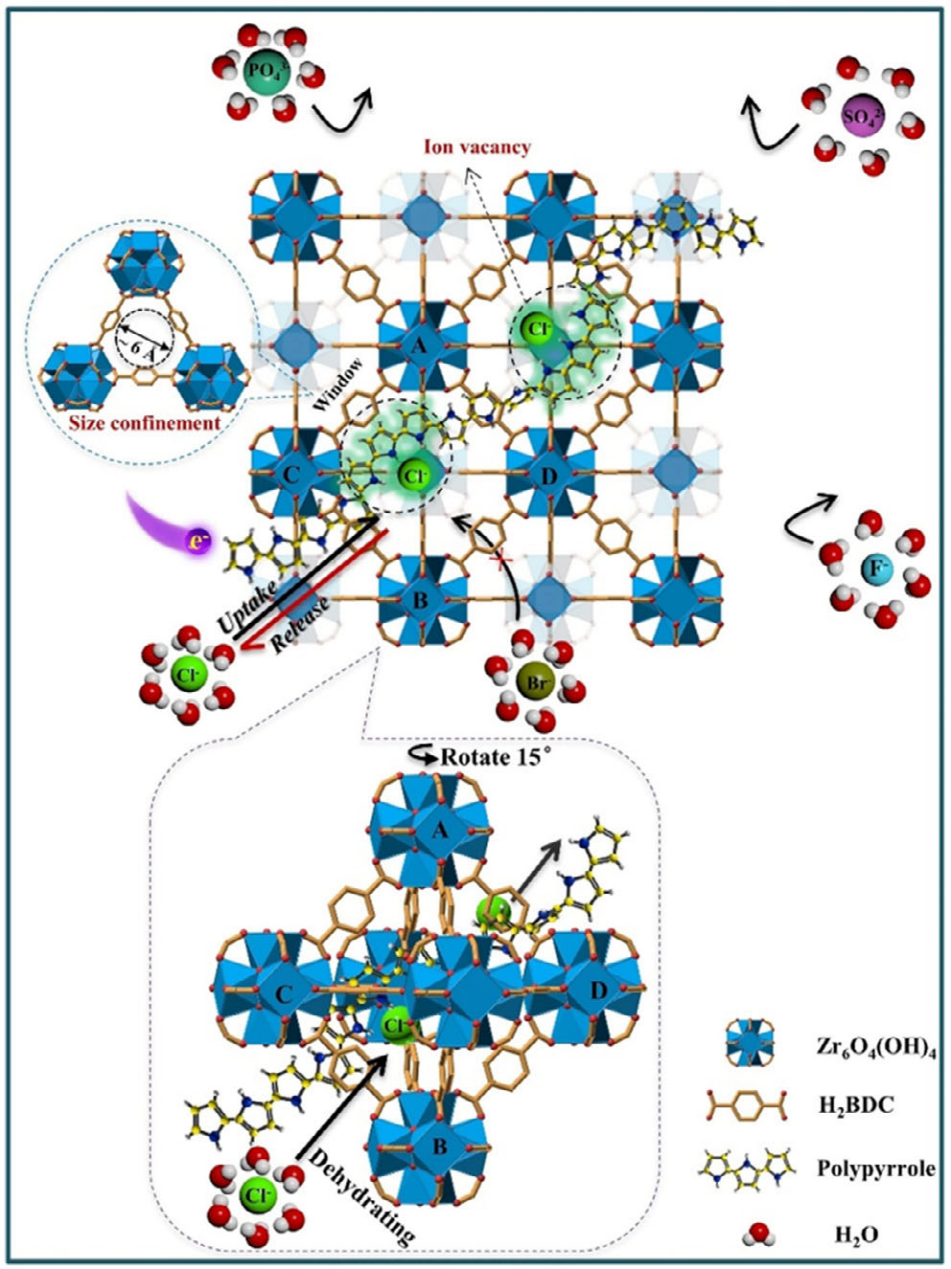
Schematic illustration of the electrochemically switched ion‐exchange process with size effect coupling with ion‐imprinted sites for the separation of Cl^−^ ions. Reproduced with permission. Reproduced with permission.^[^ ^91^ ^]^ Copyright 2021, Elsevier.

Ma et al. have devised a methodology employing a conductive chloride‐ion‐imprinted polymer (Cl–IIP) integrated within UiO‐66 to specifically segregate Cl^−^ from other ions present in wastewater.^[^ ^91^ ^]^ The fabricated conductive Cl‐IIP was threaded into the UiO‐66 via a confined polymerization approach to construct a composite film‐coated electrode known as Cl‐IIP@UiO‐66. In the presence of an E‐field, UiO‐66 functions as ion sieving channels owing to their adjustable pore sizes and well‐defined structures. Notably, the triangular window size of UiO‐66 is smaller than the hydrated ionic diameter of ions such as Cl^−^, F^−^, Br^−^, SO_4_^2−^, and PO_4_^3−^. Consequently, hydrated ions experience a dehydration process to penetrate the pores of UiO‐66, necessitating energy and escalating ion‐transfer resistance. The application of an external E‐field compensates for the energy required for dehydration, facilitating easier entry of ions into the pores. Furthermore, UiO‐66 acts as an ion filter, selectively permitting specific ions to enter while obstructing others based on their hydration energy (**Figure** **16**). Conversely, polymers like the conductive polymer PPy can exhibit cation or anion‐exchange behavior when influenced by an E‐field. Upon the application of an oxidation potential, the polymer can acquire a positive charge, enabling the incorporation of dehydrated ions into its imprinted sites. Consequently, while PPy are pivotal for facilitating ion‐exchange processes in response to E‐fields, MOFs play a vital role in selective ion separation grounded on size confinement effects. Collectively, the amalgamation of UiO‐66 structures and attributes with the utilization of an E‐field enable the targeted separation of ions. The problem that still needs to be solved is how to maintain the ion‐exchange capacity in the lower concentration of Cl^−^ solutions.

### 3.3 Molecular and Ion Diffusion/Transportation

#### 3.3.1 Molecular Diffusion/Transportation

**Figure 17:**
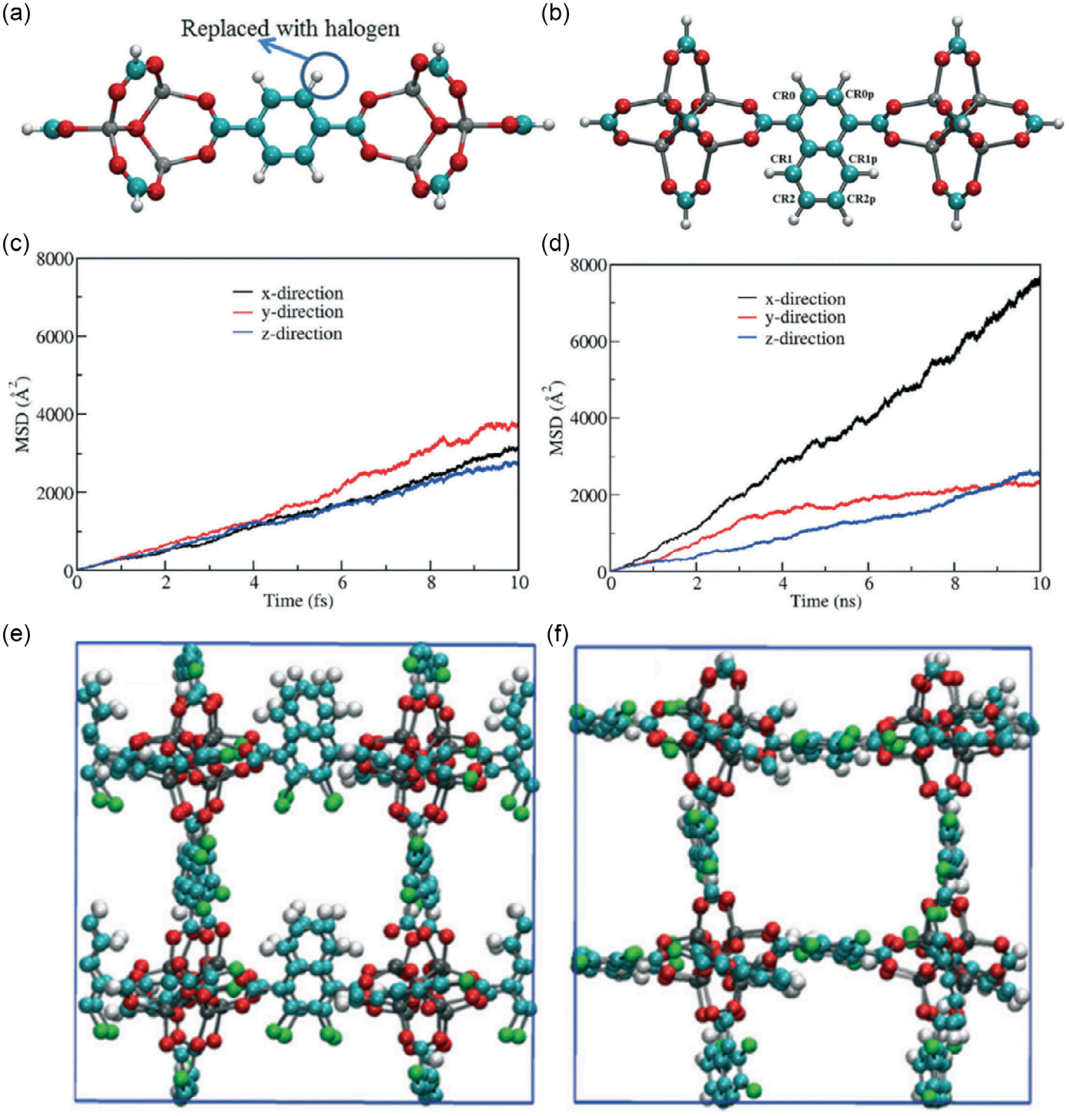
a) Model IRMOF‐1 cluster considered for dipole moment calculations with two metal nodes and a bridging ligand. b) Model IRMOF‐7 cluster with two metal nodes and a bridging ligand considered for dipole moment calculations of double Cl substitution. O, H, C, and Br atoms are shown with red, white, cyan, and green colors, respectively. MSD profiles for methane in methane‐filled CR0–CR0p‐substituted IRMOF‐7‐Cl_2_ c) without E‐field and d) with E‐field. Snapshots of CR0–CR0p‐substituted IRMOF‐7‐Cl_2_ in the x direction obtained from methane diffusion simulations e) before and f) after the E‐field is applied. Methane molecules are not shown for clarity. Reproduced with permission.^[^ ^74^ ^]^ Copyright 2018, Royal Society of Chemistry.

Halogenation of the organic linking groups of MOFs can enhance their response to the strength and direction of the E‐field, resulting in tunable gas diffusion behavior. Rotating the organic linkers by using an E‐field has demonstrated the potential to increase the diffusion of methane molecules in a specific direction while constraining their movement in other directions. Specifically, methane molecules were introduced into CR0–CR0p‐substituted (**Figure** **17**a,b) IRMOF‐7‐Cl_2_ using the grand canonical Monte Carlo simulations at 300 K and 20 atm, followed by MD simulations with and without an applied E‐field (2 V nm^−1^), respectively.^[^ ^74^ ^]^ The investigation further involved the calculation of the MSD for methane molecules in the *x*, *y*, and *z* directions with and without the application of the E‐field, as depicted in Figure 17c–d. In the absence of an E‐field, MSD values for methane molecules in the *x*, *y*, and *z* directions were nearly identical (Figure 17c), with uniform diffusion in the three directions. On the contrary, in the presence of E‐field, MSD of methane molecules increased in the x direction, decreased in the *y* direction, and remained almost unchanged in the z direction (Figure 17d). Snapshots obtained with and without applying the E‐field (Figure 17e,f) illustrated that the mobility of molecules in the x direction increased due to the rotation of ligands with about 90° angle, which allows a clear diffusion pathway but hindered the movement of methane molecules in the y direction. Notably, the mobility of methane molecules in the z direction exhibited minimal change despite ligand rotation. Importantly, the rotation of ligands induced by external E‐field and the consequent enhancement of methane diffusion were shown to be entirely reversible by altering the direction of the applied E‐field. This computational study illustrates the potential of CPMs in controlling directional molecular movement.

#### 3.3.2 Ion Diffusion/Transportation

Composed of a heulandite structure, clinoptilolite features a network of interconnected spaces filled with sizable ions and water molecules, facilitating ion swapping and reversible dehydration without altering its sturdy aluminosilicate matrix. The existence of loosely attached cations and water molecules within its pores significantly affects clinoptilolite's low‐frequency permittivity, enhancing its electrical attributes and conductivity. Salamov and colleagues documented a phenomenon where positive metal ions toward the clinoptilolite plate when subjected to a mild E‐field.^[^ ^51^ ^]^ This movement is typically observed in the presence of much stronger E‐fields. This transport process is clarified by the creation of a static negative charge in the zeolite's conductive pores adjacent to the anode, leading to an unusually intense E‐field near the anode surface. Such a potent local E‐field promotes the migration of metal ions from the anode toward the zeolite surface, where they gather, as verified by experimental data. Furthermore, the zeolite's conductivity is ascribed to the movement of positive ions through its pores, resulting in the formation of a negative charge on its surface. This negative charge, combined with the anode material's positive charge, forms a capacitor‐like structure, enabling ion migration between the anode and the zeolite. Assessing the energy balance of system is essential for understanding the stability and degree of ion migration. This study underscores the significance of zeolites in aiding mass transfer and ion desorption through their distinct structural and electrical characteristics.

**Figure 18:**
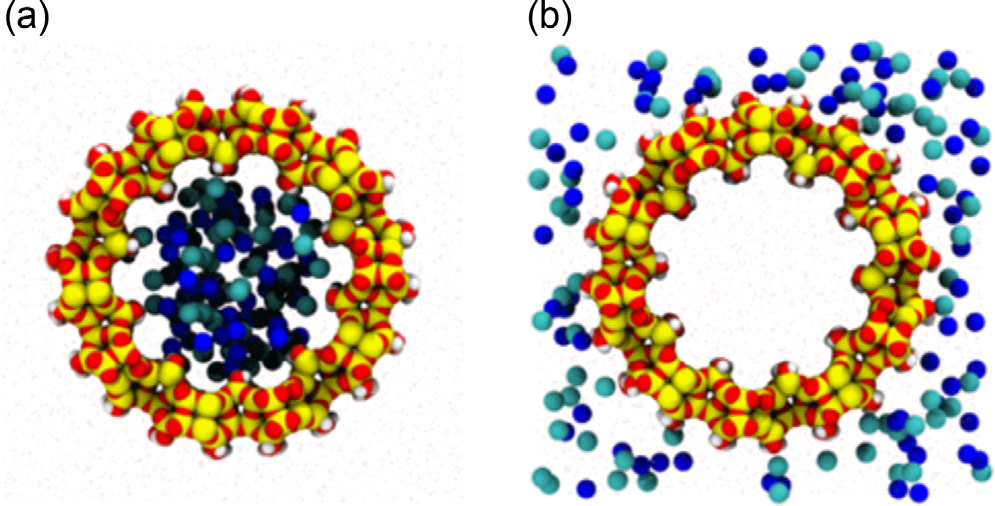
a) System configurations containing 50Na^+^ and 50Cl^−^ ions to the core of the SWZNT (S1). b) System configurations containing 150Na^+^ and 150Cl^−^ ions outside the SWZNT core (S2). Silicon shown in yellow, oxygen in red, hydrogen in white, sodium ions in blue, and chloride ions in cyan. Reproduced with permission. Reproduced with permission.^[^ ^92^ ^]^ Copyright 2022, American Chemical Society.

Hinkle conducted an MD simulation to analyze how single‐walled zeolitic nanotubes (SWZNT) behave ion transport in a NaCl water solution.^[^ ^92^ ^]^ The structure of SWZNT merged two distinct conventional frameworks: β zeolite and MFI (the structure found in ZSM‐5). The cylindrical curvature of SWZNT leads to a slight distortion of the zeolitic pores and effectively hinders the spontaneous movement of ions across the membrane in both directions (from core to bulk and vice versa), similar to the behavior observed in flat MFI zeolite systems S1 and S2 (**Figure** **18**a,b). In the specific setup S3, where 50 Na^+^ ions and 500 Cl^−^ ions are positioned inside and outside the SWZNT core, respectively, an external E‐field is applied. This setup generates a significant charge gradient across the radial direction of the SWZNT, creating a separation of positive Na^+^ ions within the SWZNT core and negative Cl^−^ ions outside. This separation leads to a transmembrane potential that serves as a reason for ion movement in S3. The E‐field triggers ion migration through the zeolitic pores in the SWZNT walls, allowing ions to cross the membrane and redistribute. Meanwhile, surface of SWZNT is hydrophilic, marked by silanol groups that bond with water molecules, fostering robust interactions with the charged ions. This affinity, combined with the unique design of the zeolitic pores, empowers the SWZNTs to efficiently manage and direct ion transport when subjected to an external E‐field. The material can be used in desalination membranes and molecular sieves. However, it is still limited to the theoretical stage.

**Figure 19:**
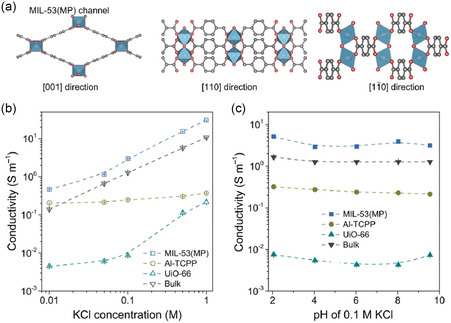
a) MIL‐53(MP) channel with medium pore form along the [001] direction, [110] direction, and [1$\bar{1}$0] direction, respectively. b) Conductivities of KCl in MOF channels measured in 0.01–1.0 M solutions. c) Conductivities of KCl in MOF channels at 0.1 M solution with pH values from 2–10. Reproduced with permission.^[^ ^60^ ^]^ Copyright 2023, Springer Nature.

Various configurations and sizes of MOF channels have been constructed to explore ion‐transport properties, encompassing the 1D MIL‐53 channel in the [001] direction, Al–TCPP (TCPP: tetrakis(4‐carboxyphenyl)porphyrin) 1D channels, 2D interlayers, and 3D UiO‐66 channels.^[^ ^60^ ^]^ Characterization studies reveal that the current value and cation mobility within MIL‐53(MP) (MP: medium pore) channels significantly surpass those observed in Al–TCPP and UiO‐66 channels. The dimensions of the 1D MIL‐53(MP) channels closely align with the diameters of hydrated monovalent cations, facilitating cation transport without substantial deformation of their hydrated shells or dehydration. Meanwhile, cations within Al–TCPP and UiO‐66 exhibit lower conductivity due to the involvement of multiple hydration and dehydration processes within the 2D and 3D channels. The conductivity of MIL‐53 channels is contingent on the solution concentration, with an increase in KCl solution concentration correlating to elevated conductivities within the MIL‐53 channels. Additionally, MOF channels devoid of functional groups demonstrate a lesser sensitivity of channel surface charge to pH variations (**Figure** **19**b,c). This indicates that pH variations do not exert a significant influence on the ion‐transport characteristics of MIL‐53 channels. Nonequilibrium MD simulations were conducted under the imposition of an external E‐field to compute ion mobility. The calculated mobility of K^+^ ions within the MIL‐53 framework marginally decreased as the strength of the E‐field intensified. Overall, the examination of how diverse solution concentrations, pH values, and E‐field strengths impact ion mobility, can offer valuable insights into ion behavior within nano‐channels and enhance their comprehension of the factors influencing ion‐transport properties in materials like MOFs.

In addition, the nanoconfinement effects of the MOF also explains the unique adsorption behavior of MOF under potential E‐field. In the construction of low‐temperature batteries, by designing a strong and flexible MOF polymer membrane (MPM) with a microstructure network, they achieved excellent fluoromethane capture at ultralow temperatures (as low as −40 °C), achieved excellent lithium‐ion conductivity, and significantly improved battery capacity compared to traditional membranes. The unique properties brought by nanoconfinement caused by the dense and continuous subnanometer micropores of MOF building blocks can be used to guide the design of ion‐conducting structures, which helps to further deepen our understanding of the performance of CPMs under E‐field.^[^ ^93^ ^]^ Cai et al. combined computational work and electrochemical measurements to study the impact of confinement effects on organic electrolytes. The study found that through the pore and structure characteristics of UiO‐66 series, the electrolyte is more stable and exhibits a quasi‐liquid‐transport state, and the conductivity is stronger, thereby enhancing the overall performance of the battery. This will provide guidance for the future design of directional transport of ions under E‐fields in nonaqueous systems.^[^ ^94^ ^]^ Due to the strong interaction characteristics of the subnanometer space of the confinement effect, the nanoporous region within the MOF can effectively shield ions and hinder anion migration, thereby enhancing Li^+^ ion migration and uniform Li^+^ ion flux specifically for electron transport. The understanding of this unique ion‐transfer behavior will provide a new perspective on ion‐confined transport in MOF under E‐field.^[^ ^95^, ^96^ ^]^

**Figure 20:**
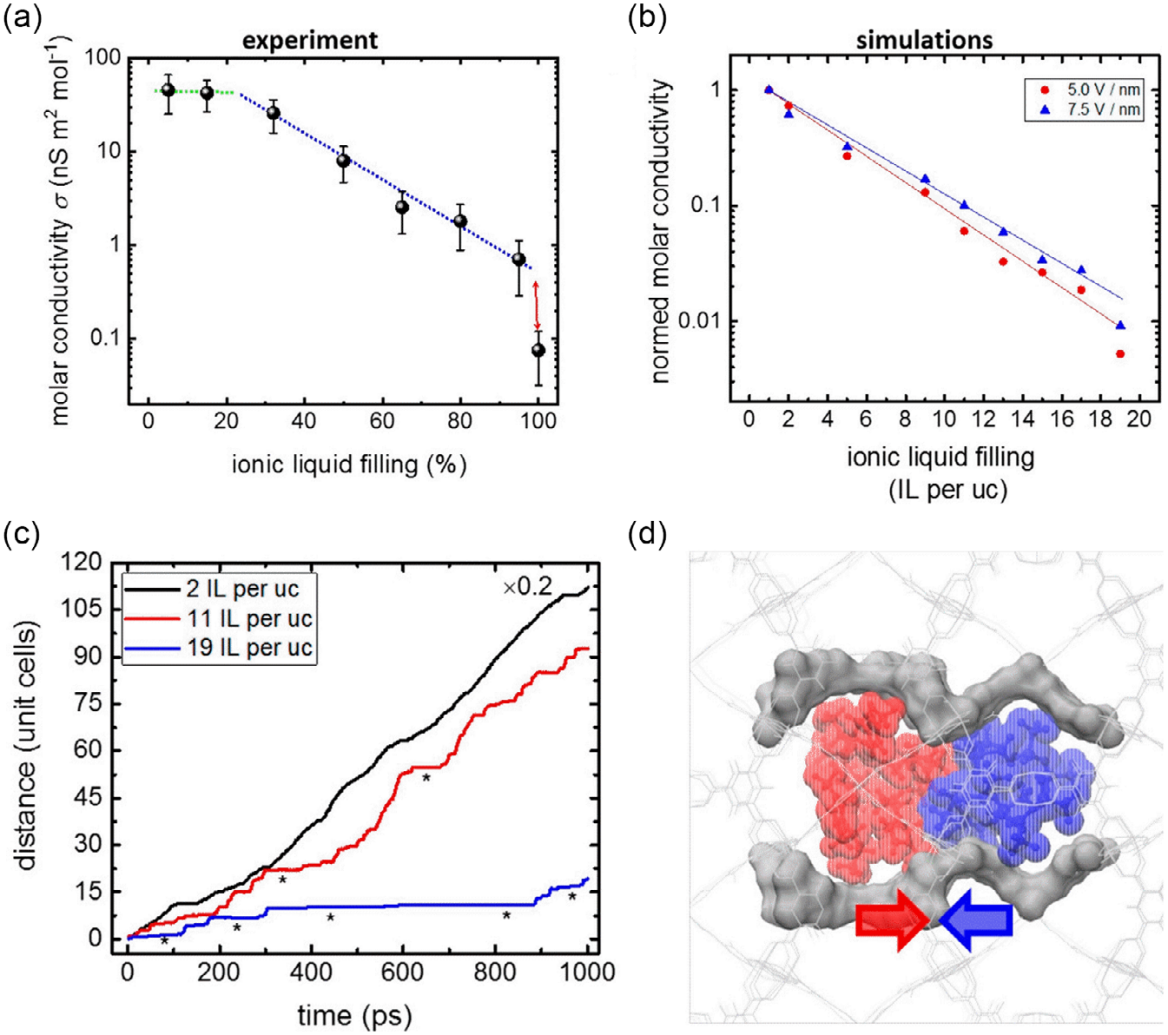
a) Experimental molar conductivity of [BMIM][NTf_2_] ionic liquid in HKUST‐1 with different pore fillings. b) Molar conductivity determined from molecular dynamics simulations with different E‐fields. c) Typical trajectories of single [BMIM] cation with different concentration. d) Sketch of a blocked MOF pore where [BMIM]^+^ (blue) and [NTf_2_]^−^ (red) try to pass simultaneously. Reproduced with permission.^[^ ^61^ ^]^ Copyright 2019, American Chemical Society.

The research involving 1‐butyl‐3‐methylimidazolium bis(trifluoromethanesulfonyl)imide ([BMIM][NTf_2_]) embedded within nanoporous HKUST‐1 delved into the ion‐transport behavior.^[^ ^61^ ^]^ At lower loadings, individual ions moved independently without obstruction, resulting in consistent conductivity. However, as the loadings increased, interactions between ions became prominent, leading to a decline in conductivity attributed to congested pathways and stationary ions (**Figure** **20**a,b). The accumulation of ions at blocked pores caused an uneven distribution of ions, further impacting ion transport. MD simulations unveiled that with an escalation in ionic liquid concentration, ions exhibited a trend of remaining immobile near high‐density pore regions, followed by bursts of movement. As loading increased, the distance covered by ions diminished. Notably, [NTf_2_]^−^ traversed shorter distances compared to [BMIM]^+^ owing to their larger mass and size, resulting in reduced diffusion rates. Furthermore, augmenting the strength of the E‐field accelerated ion movements beyond the pace observed in experiments. Interestingly, intensifying the E‐field‐strength‐induced a further decline in conductivity at elevated ionic liquid loadings. This finding underscores the role of E‐field strength in dictating the conductivity of MOF integrated with ionic liquids, offering novel insights into the electrical dynamics of ionic liquids within MOFs. The research can be further extended and more ionic liquid and MOF combinations can be studied. It will help us understand the migration of ions in the framework and help develop treatment methods for polluted ions in soil or liquid.

E‐field have been shown to induce diverse behaviors in CPMs across various environments, both in experimental and theoretical studies. These characteristics possess potential for practical applications and have been explored for use in multiple domains. **Table** **1** presented in this article outlines the applications of CPM designs discussed herein, highlighting their adaptability and potential utility in a range of applications, as induced by E‐field.

**Table 1:** Summary of E‐field‐induced CPMs adsorption behavior.

Type of CPMs	Characteristic	E‐field‐induced behavior	Potential applications	Approaches	Notes	References
Zeolite	Y zeolites	SO_2_ adsorption	Gas separation	Computational	–	[49]
Zeolite	Y zeolites/alginate hydrogel composite	FA adsorption	Drug delivery	Experimental	Solution	[79]
Zeolite	r2KCHA	Selective CO_2_/CH_4_and CO_2_/N_2_ adsorption	Gas separation	Computational and experimental	–	[37]
Zeolite	MFI zeolite membrane	Reduced permeability	Gas separation	Computational	No significant improvement	[53]
Zeolite	Zeolite 13X	H_2_O desorption	Water storage	Experimental	–	[80]
Zeolite	Na–zeolite/carbon black composite	NH_4_^+^ adsorption	Pollution treatment	Experimental	Electrode	[71]
Zeolite	HZSM‐5 composite film	Pb_2_^+^ adsorption	Pollution treatment	Experimental	Solution	[85]
Zeolite	Zeolite 13X	Cu^2+^ adsorption	Pollution treatment	Experimental	Soil	[86]
Zeolite	ZK‐4 zeolite film	NaCl/H_2_O Separation	resource exploitation	Computational	–	[89]
Zeolite	Clinoptilolite	Ion migration	Pollution treatment and resource exploitation	Experimental	Metal interface	[51]
Zeolite	SWZNT	Ion migration	Pollution treatment and resource exploitation	Computational	Solution	[92]
MOF	MIL‐53(Cr)	Selective CO_2_/CH_4_ adsorption	Gas separation	Computational	–	[57]
MOF	MIL‐53(Al)	Reduced CO_2_ capacity	Gas separation	Computational and experimental	–	[84]
MOF	*M*–MOF‐74	Selective N_2_/CH_4_ adsorption	Gas separation	Computational	–	[55]
MOF	ZIF‐8	Selective C_3_H_6_/C_3_H_8_ adsorption	Gas separation	Computational and experimental	–	[33]
MOF	MOFAC	H_2_ adsorption	Gas storage	Experimental	–	[56]
MOF	IRMOF‐7‐Cl_2_	Directed movement of guest molecules	Gas storage	Computational	–	[74]
MOF	Mg–MOF‐74	Methane molecular gates	Gas storage/separation	Computational	–	[76]
MOF	2D MOF(MON)	Gemcitabine adsorption	Drug delivery	Computational	–	[81]
MOF	NH_2_–MIL‐101(Al) composite film	PO_4_^3−^ adsorption	Pollution treatment	Computational	–	[90]
MOF	UiO‐66 composite film	Cl^−^ separation	Pollution treatment	Experimental	Electrode	[91]
MOF	MIL‐53(MP)	Ion migration	Pollution treatment and resource exploitation	Computational and experimental	Solution	[60]
MOF	[BMIM][NTf_2_]/HKUST‐1	Ion migration	Pollution treatment and resource exploitation	Computational and experimental	–	[61]
COF	B_6_N_6_	Lomustine release	Drug delivery	Computational	–	[78]
COF	Self‐assembled structure	Phenol adsorption	Pollution treatment	Computational	–	[82]
COF	TFPM–PDAN–AO and COF‐1, COF‐2	U(VI) adsorption	Resource exploitation	Experimental	–	[87, 88]

## 4 Conclusion and Outlook

**Figure 21:**
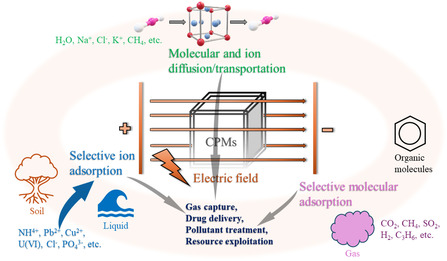
Adsorption behavior of CPMs under E‐field and its application.

In summary, our review has delineated the complex behaviors of CPMs under the influence of E‐fields, highlighting the potential of regulating adsorption properties by E‐field. The interaction of CPMs with E‐field induces noteworthy changes in their structural and electrical properties, which in turn can significantly tune their adsorption capabilities. Despite the promising outcomes observed in MOFs, COFs, and zeolites, challenges such as the need for stable structures under E‐fields and the relatively unexplored domain of CPMs within composite materials remain. To harness the full potential of CPMs in various applications, a multifaceted approach involving the adjustment of frameworks, functionalities, and system conditions is essential. The potential applications of E‐field‐induced adsorption of CPMs are intuitively represented in **Figure** **21**. Note that many CPMs require further development to improve their performance and reduce costs before they can be more effectively used in practical applications. 1) It is crucial to understand the basic principles of the behavior of CPMs under E‐field, facilitating the design of more effective materials. By employing a variety of detection methods, such as in situ electron microscopy, in situ X‐Ray diffraction analysis, and others, to thoroughly understand the changes under E‐field, the insights into the components and structural changes in CPMs can be obtained, thereby facilitating the development of potential candidates. Further consideration of material stability and the cost of large‐scale synthesis will facilitate the advancement of these materials into practical application stages. 2) Computational study can provide detailed microscopic information which is hard to get through experiments and also facilitate the preliminary validation of designed CPMs. By exploring the varied behaviors of potential CPMs under E‐field, such as framework deformation, ligand rotation, gate effects induced by the E‐field, and changes in surface charge, we are equipped with multiple approaches for material design. Selecting potential substrates based on the anticipated performance to design CPMs would be an effective strategy. However, this still necessitates extensive computational efforts and experimental validations to accumulate patterns and experience to prove in advance that CPMs with certain crystal structures or components have the desired effect under E‐field. 3) Composite materials involving CPMs, such as membranes, films, aerogels as well as electrodes, have been experimentally demonstrated to exhibit E‐field‐induced enhanced performance. This improvement is primarily attributed to the synergistic effects between CPMs and other materials under E‐field, rather than the direct response of CPMs to the E‐field itself. A thorough investigation into the adsorption mechanisms of CPMs within composite materials will aid in the more effective design of composite materials induced by E‐fields. A comprehensive analysis of how different system conditions, such as E‐field strength, temperature, and pressure, affect CPM performance is necessary. Particularly, the appropriate E‐field strength, the duration of E‐field, and the site of E‐field applied are of critical importance. Optimizing these conditions will be key to maximizing the efficiency and application of CPMs.

As we look toward the future of material science, the exploration of CPMs under E‐field emerges as a particularly promising avenue. Recent advancements in both theoretical and experimental approaches have laid a solid foundation, and now, the integration of computational modeling with cutting‐edge synthesis techniques is set to drive this field into its next phase. We anticipate that with a focused approach toward synthesizing new materials and exploring the potential of CPMs in composite materials, significant advancements will be achieved. The journey ahead promises not only to deepen our understanding of CPMs under E‐fields but also to unlock their full potential in addressing global challenges. The development of the field of adsorption on CPMs under E‐field is at a promising juncture. As we stand on the threshold of new discoveries, the potential social and scientific impact of these materials cannot be understated. The integration of CPMs into technologies for environmental cleanup, resource extraction, and healthcare, among others, offers a glimpse into a future where CPMs play important roles in solving global challenges.

## Conﬂict of Interest

The authors declare no conﬂict of interest.

## Author Contributions

**Yang Yang**: Writing—original draft: (equal). **Tianyi Zhang**: Visualization: (lead); Writing—original draft: (equal). **Tianqi Wang**: Validation: (supporting). Writing—review and editing: (supporting). **Teng Zhou**: Writing—review and editing: (supporting). **Youssef Belmabkhout**: Writing—review and editing: (supporting). **Qinfen Gu**: Formal analysis: (lead); Supervision: (lead); Writing—review and editing: (lead). **Jin Shang**: Conceptualization: (lead); Funding acquisition: (lead); Project administration: (lead); Resources: (lead); Supervision: (lead); Writing—review and editing: (lead). **Yang Yang** and **Tianyi Zhang** contributed equally to this work.

## References

[1] B. M. Weckhuysen , J. Yu , Chem. Soc. Rev. 2015, 44, 7022. 26401967 10.1039/c5cs90100f

[2] H. Furukawa , K. E. Cordova , M. O’Keeffe , O. M. Yaghi , Science 2013, 341, 1230444. 23990564 10.1126/science.1230444

[3] A. P. Côté , A. I. Benin , N. W. Ockwig , M. O’Keeffe , A. J. Matzger , O. M. Yaghi , Science 2005, 310, 1166. 16293756 10.1126/science.1120411

[4] J.‐R. Li , R. J. Kuppler , H.‐C. Zhou , Chem. Soc. Rev. 2009, 38, 1477. 19384449 10.1039/b802426j

[5] G. Li , P. Xiao , P. Webley , J. Zhang , R. Singh , M. Marshall , Adsorption 2008, 14, 415.

[6] K. Adil , Y. Belmabkhout , R. S. Pillai , A. Cadiau , P. M. Bhatt , A. H. Assen , G. Maurin , M. Eddaoudi , Chem. Soc. Rev. 2017, 46, 3402. 28555216 10.1039/c7cs00153c

[7] A. Bavykina , N. Kolobov , I. S. Khan , J. A. Bau , A. Ramirez , J. Gascon , Chem Rev 2020, 120, 8468. 32223183 10.1021/acs.chemrev.9b00685

[8] J. Yang , Y. Yang , Small 2020, 16, 1906846.

[9] J. Sculley , D. Yuan , H.‐C. Zhou , Energy Environ. Sci. 2011, 4, 2721.

[10] M. A. Gordillo , S. Saha , Comments Inorg. Chem. 2020, 40, 86.

[11] S. Afrin , M. W. Khan , E. Haque , B. Ren , J. Z. Ou , J. Colloid Interface Sci. 2022, 623, 378. 35594596 10.1016/j.jcis.2022.05.026

[12] W. Lu , Z. Wei , Z.‐Y. Gu , T.‐F. Liu , J. Park , J. Park , J. Tian , M. Zhang , Q. Zhang , T. G. Iii , M. Bosch , H.‐C. Zhou , Chem. Soc. Rev. 2014, 43, 5561. 24604071 10.1039/c4cs00003j

[13] J. Jiao , W. Gong , X. Wu , S. Yang , Y. Cui , Coord. Chem. Rev. 2019, 385, 174.

[14] B. Muir , M. Wołowiec , T. Bajda , P. Nowak , P. Czupryński , Sciendo, 48, 146, 10.1515/mipo-2017-0017.

[15] K. Xu , S. Zhang , X. Zhuang , G. Zhang , Y. Tang , H. Pang , Adv. Colloid Interface Sci. 2024, 323, 103050. 38086152 10.1016/j.cis.2023.103050

[16] L.‐D. Lin , D. Zhao , X.‐X. Li , S.‐T. Zheng , 25, 442 10.1002/chem.201803204.

[17] H.‐L. Jiang , T. A. Makal , H.‐C. Zhou , Coord. Chem. Rev. 2013, 257, 2232.

[18] R. Kitaura , K. Seki , G. Akiyama , S. Kitagawa , Angew. Chem. Int. Ed. 2003, 42, 428. 10.1002/anie.200390130 12569508

[19] J. Shang , G. Li , R. Singh , Q. Gu , K. M. Nairn , T. J. Bastow , N. Medhekar , C. M. Doherty , A. J. Hill , J. Z. Liu , P. A. Webley , J. Am. Chem. Soc. 2012, 134, 19246. 23110556 10.1021/ja309274y

[20] J. Shang , G. Li , R. Singh , P. Xiao , J. Z. Liu , P. A. Webley , J. Phys. Chem. C 2013, 117, 12841.

[21] X. Wang , R. Krishna , L. Li , B. Wang , T. He , Y.‐Z. Zhang , J.‐R. Li , J. Li , Chem. Eng. J. 2018, 346, 489.

[22] J.‐P. Zhang , H.‐L. Zhou , D.‐D. Zhou , P.‐Q. Liao , X.‐M. Chen , Nat. Sci. Rev. 2018, 5, 907.

[23] Q. Gao , J. Xu , D. Cao , Z. Chang , X.‐H. Bu , Angew. Chem. Int. Ed. 2016, 55, 15027. 10.1002/anie.201608250 27791310

[24] G. Li , J. Shang , Q. Gu , R. V. Awati , N. Jensen , A. Grant , X. Zhang , D. S. Sholl , J. Z. Liu , P. A. Webley , E. F. May , Nat. Commun. 2017, 8, 15777. 28598429 10.1038/ncomms15777 PMC5472718

[25] J. Zhao , S. H. Mousavi , G. Xiao , A. H. Mokarizadeh , T. Moore , K. Chen , Q. Gu , R. Singh , A. Zavabeti , J. Z. Liu , P. A. Webley , G. K. Li , J. Am. Chem. Soc. 2021, 143, 15195. 34516739 10.1021/jacs.1c06230

[26] S. Henke , A. Schneemann , R. A. Fischer , Adv. Funct. Mater. 2013, 23, 5990.

[27] J. Park , D. Yuan , K. T. Pham , J.‐R. Li , A. Yakovenko , H.‐C. Zhou , J. Am. Chem. Soc. 2012, 134, 99. 22148550 10.1021/ja209197f

[28] A. Modrow , D. Zargarani , R. Herges , N. Stock , Dalton Trans. 2012, 41, 8690. 22692132 10.1039/c2dt30672g

[29] Z. Wang , S. Grosjean , S. Bräse , L. Heinke , ChemPhysChem 2015, 16, 3779. 26455589 10.1002/cphc.201500829

[30] E. C. Spencer , R. J. Angel , N. L. Ross , B. E. Hanson , J. A. K. Howard , J. Am. Chem. Soc. 2009, 131, 4022. 19254021 10.1021/ja808531m

[31] P. Serra‐Crespo , A. Dikhtiarenko , E. Stavitski , J. Juan‐Alcañiz , F. Kapteijn , F.‐X. Coudert , J. Gascon , CrystEngComm 2015, 17, 276. 25722647 10.1039/C4CE00436A PMC4338503

[32] C. A. Fernandez , P. C. Martin , T. Schaef , M. E. Bowden , P. K. Thallapally , L. Dang , W. Xu , X. Chen , B. P. McGrail , Sci. Rep. 2014, 4, 6114. 25135307 10.1038/srep06114 PMC4137262

[33] A. Knebel , B. Geppert , K. Volgmann , D. I. Kolokolov , A. G. Stepanov , J. Twiefel , P. Heitjans , D. Volkmer , J. Caro , Science 2017, 358, 347. 29051376 10.1126/science.aal2456

[34] S. Yao , M. Zheng , S. Wang , T. Huang , Z. Wang , Y. Zhao , W. Yuan , Z. Li , Z. L. Wang , L. Li , Adv. Funct. Mater. 2022, 32, 2209142.

[35] Y. Tian , A. Stroppa , Y. Chai , L. Yan , S. Wang , P. Barone , S. Picozzi , Y. Sun , Sci. Rep. 2014, 4, 6062. 25317819 10.1038/srep06062 PMC5377545

[36] Y. Ma , Y. Wang , J. Cong , Y. Sun , Phys. Rev. Lett. 2019, 122, 255701. 31347892 10.1103/PhysRevLett.122.255701

[37] K. Chen , Z. Yu , S. H. Mousavi , R. Singh , Q. Gu , R. Q. Snurr , P. A. Webley , G. K. Li , Nat. Commun. 2023, 14, 5479. 37673916 10.1038/s41467-023-41227-4 PMC10482906

[38] K. Koseoglu , I. Karaduman , M. Demir , M. Ozer , S. Acar , B. G. Salamov , Superlattices Microstruct. 2015, 81, 97.

[39] S. O. Koc , K. Koseoglu , S. Galioglu , B. Akata , B. G. Salamov , Microporous Mesoporous Mater. 2016, 223, 18.

[40] D. Fu , W. Zhang , H. Cai , Y. Zhang , J. Ge , R. Xiong , S. D. Huang , T. Nakamura , Angew. Chem. Int. Ed. 2011, 50, 11947. 10.1002/anie.201103265 22012691

[41] P. Jain , A. Stroppa , D. Nabok , A. Marino , A. Rubano , D. Paparo , M. Matsubara , H. Nakotte , M. Fiebig , S. Picozzi , E. S. Choi , A. K. Cheetham , C. Draxl , N. S. Dalal , V. S. Zapf , NPJ Quant. Mater. 2016, 1, 16012.

[42] E. B. Winston , P. J. Lowell , J. Vacek , J. Chocholoušová , J. Michl , J. C. Price , Phys. Chem. Chem. Phys. 2008, 10, 5188. 18728858 10.1039/b808104b

[43] J. Zhao , J. Xu , S. Han , Q. Wang , X. Bu , Adv. Mater 2017, 29, 1606966. 10.1002/adma.201606966 28401592

[44] P. Shao , J. Li , F. Chen , L. Ma , Q. Li , M. Zhang , J. Zhou , A. Yin , X. Feng , B. Wang , Angew. Chem. Int. Ed. 2018, 57, 16501. 10.1002/anie.201811250 30334322

[45] Z.‐F. Cai , G. Zhan , L. Daukiya , S. Eyley , W. Thielemans , K. Severin , S. De Feyter , J. Am. Chem. Soc. 2019, 141, 11404. 31280563 10.1021/jacs.9b05265

[46] S.‐M. Wang , Y.‐H. Jin , L. Zhou , K.‐H. Wang , H. J. Kim , L. Liu , E. Kim , Z. Han , ACS Appl. Mater. Interfaces 2023, 15, 542, acsami.3c11948. 10.1021/acsami.3c11948 37976415

[47] J. P. Dürholt , B. F. Jahromi , R. Schmid , ACS Cent. Sci. 2019, 5, 1440. 31482127 10.1021/acscentsci.9b00497 PMC6716137

[48] R. Schmid , ACS Cent. Sci. 2017, 3, 369. 28573197 10.1021/acscentsci.7b00162 PMC5445533

[49] Y. Sabahi , M. Razmkhah , F. Moosavi , Mater. Today Commun. 2022, 30, 103045.

[50] A. I. Prilipko , V. G. Il'in , V. P. Shabel'nikov , V. G. Golovatyi , N. V. Turutina , Theor. Exp. Chem. 1989, 24, 600.

[51] V. I. Orbukh , N. N. Lebedeva , Z. A. Agamaliev , G. M. Eivazova , B. G. Salamov , Tech. Phys. 2020, 65, 312.

[52] E. C. De Lara , R. Kahn , R. Seloudoux , J. Chem. Phys. 1985, 83, 2646.

[53] W. Jia , S. Murad , J. Chem. Phys. 2005, 122, 234708. 16008474 10.1063/1.1930829

[54] S. Zhou , Y. Wei , L. Li , Y. Duan , Q. Hou , L. Zhang , L.‐X. Ding , J. Xue , H. Wang , J. Caro , Sci. Adv. 2018, 4, eaau1393. 30410983 10.1126/sciadv.aau1393 PMC6218190

[55] H. Kim , J. Kim , RSC Adv. 2022, 12, 23396. 36090418 10.1039/d2ra04216a PMC9390010

[56] S. Xie , J.‐Y. Hwang , X. Sun , S. Shi , Z. Zhang , Z. Peng , Y. Zhai , J. Power Sources 2014, 253, 132.

[57] A. Ghoufi , K. Benhamed , L. Boukli‐Hacene , G. Maurin , ACS Cent. Sci. 2017, 3, 394. 28573200 10.1021/acscentsci.6b00392 PMC5445524

[58] L. Lyu , H. Wu , L. Li , Y. Wei , H. Wang , Chem. Ing. Tech. 2022, 94, 119.

[59] C. Du , Y. Zhang , Z. Zhang , L. Zhou , G. Yu , X. Wen , T. Chi , G. Wang , Y. Su , F. Deng , Y. Lv , H. Zhu , Chem. Eng. J. 2022, 431, 133932.

[60] X. Li , G. Jiang , M. Jian , C. Zhao , J. Hou , A. W. Thornton , X. Zhang , J. Z. Liu , B. D. Freeman , H. Wang , L. Jiang , H. Zhang , Nat. Commun. 2023, 14, 286. 36653373 10.1038/s41467-023-35970-x PMC9849445

[61] A. B. Kanj , R. Verma , M. Liu , J. Helfferich , W. Wenzel , L. Heinke , Nano Lett. 2019, 19, 2114. 30830791 10.1021/acs.nanolett.8b04694

[62] J. Widakdo , H. F. M. Austria , T. M. Subrahmanya , E. Suharyadi , W.‐S. Hung , C.‐F. Wang , C.‐C. Hu , K.‐R. Lee , J.‐Y. Lai , J. Mater. Chem. A 2022, 10, 16743.

[63] A. Jayakumar , V. K. Jose , J.‐M. Lee , Small Methods 2020, 4, 1900735.

[64] K. Y. Foo , B. H. Hameed , J. Hazard. Mater. 2009, 170, 552. 19501461 10.1016/j.jhazmat.2009.05.057

[65] R. de Mello , A. J. Motheo , C. Sáez , M. A. Rodrigo , Curr. Opin. Electrochem. 2022, 36, 101167.

[66] B. Soltabayev , H. Hilal Kurt , S. Acar , B. G. Salamov , Journal of Elec Materi 2019, 48, 6910.

[67] S. M. Kuznicki , V. A. Bell , S. Nair , H. W. Hillhouse , R. M. Jacubinas , C. M. Braunbarth , B. H. Toby , M. Tsapatsis , Nature 2001, 412, 720. 11507636 10.1038/35089052

[68] T.‐C. Wei , H. W. Hillhouse , J. Phys. Chem. B 2006, 110, 13728. 16836317 10.1021/jp061037u

[69] J. Dong , Z. Xu , S. Yang , S. Murad , K. R. Hinkle , Curr. Opin. Chem. Eng. 2015, 8, 15.

[70] S. M. Hosseini , S. Rafiei , A. R. Hamidi , A. R. Moghadassi , S. S. Madaeni , Desalination 2014, 351, 138.

[71] X. He , W. Chen , F. Sun , Z. Jiang , B. Li , X. Li , L. Lin , Environ. Sci. Technol. 2023, 57, 8828. 37246552 10.1021/acs.est.3c02286

[72] A. Alamdari , R. Karimzadeh , Catalysts 2018, 8, 270.

[73] A. Alamdari , R. Karimzadeh , React. Kinet. Mech. Catal. 2018, 123, 723.

[74] S. Namsani , A. Ozgur Yazaydin , Mol. Syst. Des. Eng. 2018, 3, 951.

[75] A. L. Kolesnikov , Y. A. Budkov , J. Möllmer , M. G. Kiselev , R. Gläser , J. Phys. Chem. C 2019, 123, 10333.

[76] B. Tam , O. Yazaydin , J. Mater. Chem. A 2017, 5, 8690.

[77] G. Zhan , Z.‐F. Cai , M. Martínez‐Abadía , A. Mateo‐Alonso , S. De Feyter , J. Am. Chem. Soc. 2020, 142, 5964. 32196321 10.1021/jacs.0c01270

[78] M. Heidari , M. Solimannejad , J. Inorg. Organomet. Polym. 2022, 32, 4216.

[79] N. Paradee , A. Sirivat , Mol. Pharmaceutics 2016, 13, 155. 10.1021/acs.molpharmaceut.5b00592 26561875

[80] G. Roussy , A. Zoulalian , M. Charreyre , J. M. Thiebaut , J. Phys. Chem. 1984, 88, 5702.

[81] A. Haqyar , H. Raissi , F. Farzad , H. Hashemzadeh , Inorg. Chem. Commun. 2022, 138, 109281.

[82] A. Ghahari , H. Raissi , S. Pasban , F. Farzad , NPJ Clean Water 2022, 5, 28.

[83] A. Ghahari , H. Raissi , F. Farzad , J. Taiwan Inst. Chem. Eng. 2021, 125, 15.

[84] K. Chen , R. Singh , J. Guo , Y. Guo , A. Zavabeti , Q. Gu , R. Q. Snurr , P. A. Webley , G. K. Li , ACS Appl. Mater. Interfaces 2022, 14, 13904. 35276036 10.1021/acsami.1c24335

[85] X. Du , D. Zhang , X. Ma , W. Qiao , Z. Wang , X. Hao , G. Guan , Electrochim. Acta 2018, 282, 384.

[86] O. Ursini , E. Lilla , R. Montanari , J. Hazard. Mater. 2006, 137, 1079. 16716501 10.1016/j.jhazmat.2006.03.070

[87] C.‐R. Zhang , J.‐X. Qi , W.‐R. Cui , X.‐J. Chen , X. Liu , S.‐M. Yi , C.‐P. Niu , R.‐P. Liang , J.‐D. Qiu , Sci. China Chem. 2023, 66, 562.

[88] S. Yang , J. Yin , Q. Li , C. Wang , D. Hua , N. Wu , J. Hazard. Mater. 2022, 429, 128315. 35077974 10.1016/j.jhazmat.2022.128315

[89] S. Murad , J. Lin , Ind. Eng. Chem. Res. 2002, 41, 1076.

[90] R. Liu , Y. Sui , X. Wang , Chem. Eng. J. 2019, 371, 903.

[91] W. Ma , X. Du , M. Liu , F. Gao , X. Ma , Y. Li , G. Guan , X. Hao , Chem. Eng. J. 2021, 412, 128576.

[92] K. R. Hinkle , J. Phys. Chem. C 2022, 126, 6803.

[93] G. Cai , Y. Yin , D. Xia , A. A. Chen , J. Holoubek , J. Scharf , Y. Yang , K. H. Koh , M. Li , D. M. Davies , M. Mayer , T. H. Han , Y. S. Meng , T. A. Pascal , Z. Chen , Nat. Commun. 2021, 12, 3395. 34099643 10.1038/s41467-021-23603-0 PMC8184934

[94] G. Cai , A. A. Chen , S. Lin , D. J. Lee , K. Yu , J. Holoubek , Y. Yin , A. U. Mu , Y. S. Meng , P. Liu , S. M. Cohen , T. A. Pascal , Z. Chen , Nano Lett. 2023, 23, 7062. 37522917 10.1021/acs.nanolett.3c01825

[95] A. U. Mu , G. Cai , Z. Chen , Adv. Sci. 2024, 11, 2305280. 10.1002/advs.202305280 PMC10787081 37946699

[96] R. Lv , C. Luo , B. Liu , K. Hu , K. Wang , L. Zheng , Y. Guo , J. Du , L. Li , F. Wu , R. Chen , Adv. Mater. 2024, 36, 2400508. 10.1002/adma.202400508 38452342

